# Phylogenomics of Ligand-Gated Ion Channels Predicts Monepantel Effect

**DOI:** 10.1371/journal.ppat.1001091

**Published:** 2010-09-09

**Authors:** Lucien Rufener, Jennifer Keiser, Ronald Kaminsky, Pascal Mäser, Daniel Nilsson

**Affiliations:** 1 Novartis Centre de Recherche Santé Animale, St. Aubin, Switzerland; 2 Institute of Cell Biology, University of Bern, Bern, Switzerland; 3 Swiss Tropical and Public Health Institute, Basel, Switzerland; 4 University of Basel, Basel, Switzerland; Trudeau Institute, United States of America

## Abstract

The recently launched veterinary anthelmintic drench for sheep (Novartis Animal Health Inc., Switzerland) containing the nematocide monepantel represents a new class of anthelmintics: the amino-acetonitrile derivatives (AADs), much needed in view of widespread resistance to the classical drugs. Recently, it was shown that the ACR-23 protein in *Caenorhabditis elegans* and a homologous protein, MPTL-1 in *Haemonchus contortus*, are potential targets for AAD action. Both proteins belong to the DEG-3 subfamily of acetylcholine receptors, which are thought to be nematode-specific, and different from those targeted by the imidazothiazoles (e.g. levamisole). Here we provide further evidence that *Cel*-ACR-23 and *Hco*-MPTL-1-like subunits are involved in the monepantel-sensitive phenotype. We performed comparative genomics of ligand-gated ion channel genes from several nematodes and subsequently assessed their sensitivity to anthelmintics. The nematode species in the *Caenorhabditis* genus, equipped with ACR-23/MPTL-1-like receptor subunits, are sensitive to monepantel (EC_50_<1.25 µM), whereas the related nematodes *Pristionchus pacificus* and *Strongyloides ratti*, which lack an ACR-23/MPTL-1 homolog, are insensitive (EC_50_>43 µM). Genome sequence information has long been used to identify putative targets for therapeutic intervention. We show how comparative genomics can be applied to predict drug sensitivity when molecular targets of a compound are known or suspected.

## Author Summary

Increased use of anthelmintics has contributed to the emergence of drug-resistant nematodes, causing serious problems for more than one billion sheep worldwide. The last class of compounds indicated for livestock was introduced 28 years ago. Recently, however, Novartis AH developed a new anthelmintic active against drug-resistant nematodes of sheep, the amino-acetonitrile derivative (AAD) monepantel. We have previously indirectly shown that the AADs have a novel mode of action involving acetylcholine receptor subunits: the ACR-23 protein in *Caenorhabditis elegans* and a homologous protein, MPTL-1 in *Haemonchus contortus*. To better understand the mode of action of the AADs, we performed comparative genomics of all ligand-gated ion channel genes from a range of organisms, including members from all nematode clades. We confirmed that MPTL-1 belongs to a unique, nematode-specific sub-family of receptor subunits. We also found that some nematode species lack ACR-23/MPTL-1 and predicted them to be monepantel insensitive. We challenged this hypothesis in a panel of drug tests: several species of *Caenorhabditis* nematodes equipped with ACR-23/MPTL-1-like receptor subunits were found susceptible to monepantel, whereas *Pristionchus pacificus*, closely related to these worms but lacking an ACR-23/MPTL-1 homolog, was tolerant. The parasitic nematode *Strongyloides ratti*, which has only a remote homolog of DES-2 and ACR-23/MPTL-1, was also tolerant to monepantel. This confirms our prediction and highlights how comparative genomic data can be used to predict a drug effect.

## Introduction

Nematode parasites of sheep represent one of the major constraints in the wool, meat and milk industries world wide [1]. The gastro-intestinal parasite *Haemonchus contortus*, in particular, causes substantial losses. In the mid-1950s, the existence of anthelmintic resistant worm populations came to light with the failure of phenothiazine against *Haemonchus* [2]. Since then, nematode populations resistant to the three classical groups of anthelmintics, i.e. the benzimidazoles, imidazothiazoles and macrocyclic lactones, and combinations of these have been described [3]–[7]. Recently, the amino-acetonitrile derivatives (AADs) have been reported as a new class of synthetic anthelmintics active against gastro-intestinal nematodes of sheep [8] and a first drug from this family, monepantel, was, at the time of writing licensed to market in Australasia, Europe and Latin America (ZOLVIX; Novartis Animal Health Inc., Switzerland; [9]). Investigations to understand the mode of action of the AADs in *Caenorhabditis elegans* have been performed using chemical mutagenesis and gene mapping via a genetic recombination approach. Out of 44 isolated resistant alleles, 36 were mapped to a single gene, *acr-23*, designating it as a major contributor to the AAD response in *C. elegans* [8]. A further study on mutant *H. contortus* isolates identified the gene *monepantel-1* (*Hco-mptl-1*) as a major target candidate for AADs action in this species [8], [10]. Both *Cel-acr-23* and *Hco-mptl-1* are predicted to encode a nicotinic acetylcholine receptor (nAChR) subunit. These belong to the superfamily of ligand-gated ion channel (LGIC) subunits. These are the modular components with the ability to form a large number of channels with different properties through heteromultimerisation (see e.g. [11]), as all characterised LGIC function as penta- or tetramers. They provide many important drug and toxin targets: levamisole and pyrantel act as agonists of nAChR [12], paraherquamide as a competitive antagonist of these channels [13] and ivermectin modulates glutamate-gated chloride channels [12], [14], [15]. ACR-23 and MPTL-1 are members of the DEG-3 subfamily of acetylcholine receptor subunit genes and distinct from those targeted by imidazothiazoles [16], [17]. Members of this subfamily have so far only been found in nematodes and no cross-resistance between the AADs and the imidazothiazoles have been documented [8].

**Figure 1:**
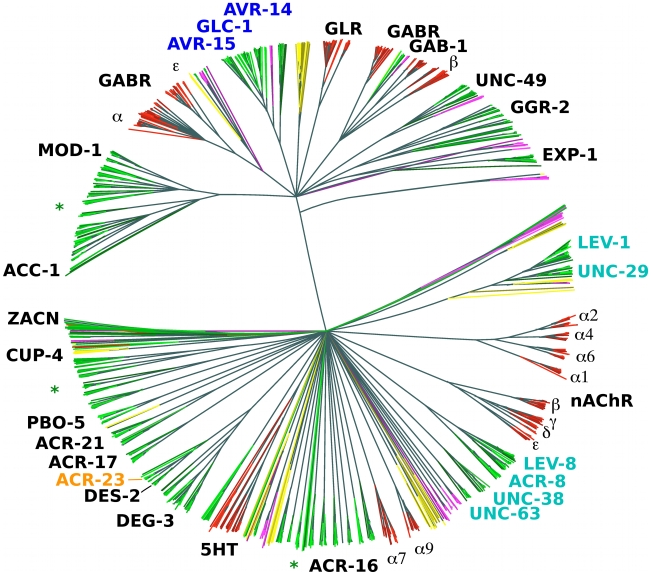
Phylogenetic tree based on the LBD region of putative LGIC genes. NJ tree (clustalw) from the LBD region of conceptually translated putative LGIC genes as detected with Genewise after an initial Blast screen with the 210 seeds (see Materials and Methods) - 1426 sequences in total. Thousand bootstrap iterations were performed and branches below 50% bootstrap support were collapsed. Nematode sequences are shown in shades of green, platyhelminthes yellow, insect purple and vertebrate red. Some *C. elegans* and human subunits are labelled, and the labels for proteins involved in drug susceptibility are coloured: levamisole - cyan, monepantel - orange and ivermectin – blue. Green asterisks indicate branches that similar to these latter appear broad and nematode specific and could be attractive for further investigation as targets for other compounds.

All animals appear to have about the same number of nAChR α subunits (around 16), with exception of the nematodes [18]. Among the completely sequenced genomes from animals, those with the highest (*C. elegans*) and smallest (*Brugia malayi*) numbers of such genes are both from the nematodes [19]. The reason for the variation in numbers of nAChR α subunits and other LGIC subunits is not clear. However, certain LGIC subunits form heteromultimeric channels that provide prominent anthelmintic-specific drug targets (highlighted in Figure 1), in particular for ivermectin (AVR-14, AVR-15, GLC-1), levamisole (UNC-38, UNC-29, UNC-63, LEV-1, LEV-8) and monepantel (MPTL-1). Several new, draft nematode genomes as well as pre-publication quality assemblies are now available from ongoing or recently finished sequencing projects (see Table 1).

**Table 1:** Genome sequences included in tree construction from indicated phylum.

Species	Phylum	Lifestyle	LGICs	Genome (Mbp)	Db Unit Mean (bp)	Genome status	Source/Ref
*Trichinella spiralis*	Nematoda Clade I	P, G	24	71	6117	Pre-finishing	GSC, WUSTL
*Ascaris suum*	Nematoda Clade III	P, G	5	230	1718^a^	In progress	WTSI/NematodeNet
*Brugia malayi*	Nematoda Clade III	P, G	24	∼100	3266^b^	Draft	[39]
*Strongyloides ratti*	Nematoda Clade IVa	F, G/H	47	?	3825^a^	In progress	WTSI
*Meloidogyne hapla*	Nematoda Clade IVb	P, Pg/G	32	54	15108^b^	Draft	[40]
*Meloidogyne incognita*	Nematoda Clade IVb	P, Pg	24	86	8607	Draft	[41]
*Caenorhabditis brenneri*	Nematoda Clade V	B, G	101	150	40037^b^	In progress	GSC, WUSTL
*Caenorhabditis briggsae*	Nematoda Clade V	B, G/H	81	105	99356^b^	Complete	[42]
*Caenorhabditis elegans*	Nematoda Clade V	B, G/H	84	100	99727^b^	Complete	[43]
*Caenorhabditis japonica*	Nematoda Clade V	B, G	69	∼100?	20168^b^	In progress	GSC, WUSTL
*Caenorhabditis remanei*	Nematoda Clade V	B, G	87	150	10780^b^ ^a^	Pre-finishing	GSC, WUSTL
*Haemonchus contortus*	Nematoda Clade V	P, G	41	60	4991	In progress	WTSI
*Heterorhabditis bacteriophora*	Nematoda Clade V	F, G/H	61	∼110	14630^a^	In progress	GSC, WUSTL
*Nippostrongylus brasiliensis*	Nematoda Clade V	P,G	0	?	492^a^	In progress	WTSI
*Pristionchus pacificus*	Nematoda Clade V	F, H	54	170	9537	Draft	[44]
*Teladorsagia circumcincta*	Nematoda Clade V	P, G	2	?	1994	In progress	WTSI
*Echinococcus multilocularis*	Platyhelm Cestoda	P, G/H	13	150	5585	In progress	WTSI
*Schistosoma mansoni*	Platyhelm Trematoda	P, G	13	270	7455	Draft	[45]
*Schmidtea mediterranea*	Platyhelm Turbellaria	O, H	46	?	9113^b^	Unpublished	GSC, WUSTL
*Danio rerio*	Vertebrata	O, G	56	1500	58949^b^	Draft	WTSI, community
*Takifugu rubripes*	Vertebrata	O, G	57	400	25094^b^	Draft	[46]
*Gasterosteus aculeatus*	Vertebrata	O, G	64	460	74917^b^	In progress	Broad
*Xenopus tropicalis*	Vertebrata	O, G	38	1500?	47478^b^	In progress	JGI
*Gallus gallus*	Vertebrata	O, G	30	1050?	99694^b^	Draft	[47]
*Homo sapiens sapiens*	Vertebrata	O, G	36	2865	99841^b^	Draft	[48]
*Bos taurus*	Vertebrata	He, G	37	2870	73885^b^	Draft	[49]
*Canis familiaris*	Vertebrata	C, G	36	2041	99919^b^	Draft	[50]
*Felis catus*	Vertebrata	C, G	21	∼3000	18263^b^	In progress	Broad/Agencourt
*Drosophila melanogaster*	Diptera	O, G	21	180	99631^b^	Draft	[51]
*Aedes aegypti*	Diptera	O, G	16	1376	79749^b^	Draft	[52]
*Anopheles gambiae*	Diptera	O, G	20	260	99913^b^	Draft	[53]
*Culex quinquefasciatus*	Diptera	O, G	19	540	75385^b^	In progress	Broad/JCVI
*Ixodes scapularis*	Chelicerata	O, G	14	2000?	4726^b^	In progress	Broad/JCVI

The lifestyle of each species is indicated as Omnivore, O; Parasite, P; Facultative parasite, F; Herbivore, He; Bacteriovore, B; or Carnivore, C; followed by reproductive modes Gonochoristic, G; Parthenogenetic, Pg; or Hermaphrodite, H. LGIC denotes the number of genes with LBDs (PFAM PF02931.15) found in the respective genomes. Approximate genome sizes are given in million base pairs (Mbp) and mean contig sizes in bp.

a the analysis was performed on contig rather than supercontig level.

b contigs were artificially truncated to 100 kbp segments with 2 kbp overlap. Published genomes or draft genomes are give with citations, and sequencing organisation otherwise (GenBank: http://www.ncbi.nlm.nih.gov; Ensembl: http://www.ensembl.org; WTSI: http://www.sanger.ac.uk; nematode.net: http://www.nematode.net; GSC/WUSTL: http://genome.wustl.edu; Broad: http://www.broadinstitute.org; WormBase: http://www.wormbase.org; JGI: http://www.jgi.doe.gov).

To learn what parts of the LGIC superfamily are unique to nematodes and in consideration of much new sequence information, we constructed a simple phylogenomic pipeline to further understand the mechanisms behind the action of monepantel. We explored the LGIC superfamily by *in silico* searches, and while we found a considerable number of tentative new family members since the last such survey was made [19], the DEG-3-subfamily remains nematode specific. In an *in vitro* drug assay we further show that susceptibility to the AADs directly follows the presence of ACR-23 or MPTL-1 homologs in the genomes from the nematodes investigated.

## Materials and Methods

### Genome analysis

Genome data in the form of contig or supercontig DNA sequence fasta files were downloaded from GenBank (NCBI), Ensembl, WTSI, nematode.net, GSC/WUSTL, Broad and WormBase (all attributed and referenced in Table 1). Sequences from genomes with long contiguous sequences were artificially divided into 100 kb segments (indicated by asterisks in Table 1). Seed sequences were obtained as peptide fasta files from WormBase [20] and Uniprot [21].

A Blast [22] screen with the seed sequences as queries against the genomic sequence databases was performed. Only contigs with hits (E<0.1) were searched by Genewise [23] with the PFAM [24] motifs LGIC_LBD (PF02931.15) and LGIC_MEMB (PF02932.8) for global scoring (ls). Splice sites were considered using the Genewise-provided worm gene model. The seed peptide sequences were searched using the same PFAM profiles but with hmmsearch from the hmmer2 package (by Eddy, http://hmmer.janelia.org). The protein domains, conceptually translated from DNA or directly from the seed proteins, that exhibited E-values below the trusted E-value cut-off were aligned and an nj tree (bootstrap 1000 iterations) was constructed with clustalw [25]. These steps were automated in bash and Perl using tools from the EMBOSS package [26] and executed on LINUX computers using less than 1.5Gb RAM. Trees were visualised with Dendroscope [27] and HyperTree [28]. For Figure 1, furcations with bootstrap support below 50% were fused in jtreeview (Frickey, Lupas http://www.eb.tuebingen.mpg.de/departments/1-protein-evolution/software/jtreeview/). Co-segregation with known named seed sequences in the bootstrapped tree was used for assigning putative identity to homologous genes. Trees based on available, confirmed or predicted, full-length protein sequences (WormBase WS195 *C. brenneri*, *C. elegans*, *C. briggsae*, *C. japonica*, *C. remanei*, *Pristionchus pacificus* and *B. malayi*) were also constructed. The same seed sequences were used to pick predicted genes with a blastp similarity (E<0.01) for inclusion in a profile search and tree construction using the aforementioned methods.

### Nematode strains

Nematode strains *C. briggsae* AF16, *C. brenneri* PB2801, *C. remanei* PB4641, *C. japonica* DF5081 and *P. pacificus* PS312 as well as the mutants VC1598: *Cel-acr-20(ok1849)/mT1 II*; *+/mT1[dpy-10(e128)] III*, NC293: *Cel-acr-5(ok180) III*, TU1803: *Cel-deg-3(u662) Cel-des-2(u695) V* and RB1226: *Cel-acr-18(ok1285) V* were obtained from the *Caenorhabditis* Genetics Center (CGC), Minneapolis, USA, which is funded by the NIH National Center for Research Resources (NCRR). *Caenorhabditis elegans* Bristol N2 and AP134: *Cel-acr-23 (cb27) V* [8] were kind gifts from Prof. Alessandro Puoti, University of Fribourg. Nematodes were maintained at 20°C on Nematode Growth Medium (NGM) plates (3 g NaCl, 17 g Agar, 2.5 g peptone in 975 ml H_2_O, autoclaved, added 1 ml cholesterol (Sigma) prepared to 5 mg/ml in EtOH, 1 ml M CaCl_2_, 1 ml M MgSO_4_ and 25 ml KPO_4_-buffer), and inoculated with *E. coli* OP50, and transferred every 3 days.

*Strongyloides ratti* L3 were obtained from the feces of infected rats following standardized procedures based on the Baermann technique at the Swiss Tropical and Public Health Institute. Freshly harvested *S. ratti* L_3_ were washed 3 times with PBS buffer and used immediately for in vitro drug testing. The species of nematodes used in the in vitro drug test was confirmed by a PCR targeting the 18S rRNA region of *C. elegans*, *C. briggsae*, *C. remanei*, *C. brenneri*, *C. japonica* and *P. pacificus*. Using the forward primer SSU18A and the reverse primer SSU26R [29] (Supplementary Table S1), a ∼950 bp fragment was amplified using FastStart High Fidelity PCR system (Roche). The reaction conditions were: 95°C for 10 min without Taq polymerase; 95°C for 2 min; 35 cycles of [95°C for 30 sec; 52°C for 30 sec; 72°C for 1 min 10 sec]; 72°C for 10 min. PCR products were purified using the Wizard SV PCR Clean-Up kit (Promega) and sequenced in both directions with SSU18A and SSU26R at Microsynth AG (http://www.microsynth.ch). Sequence quality check and assembly was done using 4Peaks (by A. Griekspoor and T. Groothuis; http://mekentosj.com) and a nucleotide blast was made on-line (NCBI) against the nucleotide collection (nt).

### Drug test

Ivermectin and AAD-1566 were provided by Novartis Animal Health, Centre de Recherche Santé Animale, Fribourg, Switzerland. The compounds were diluted in pure DMSO to 10 mM and 250 mM, respectively. Appropriate dilutions of drugs were placed at the bottom of wells in 24-well plates and 1 ml NGM was added per well. The first well in each row served as a control with 1% DMSO. The plates were well shaken, allowed to dry at RT for several days, then inoculated with 10 µL *E. coli* OP50 and incubated at 37°C overnight. Eggs were purified from adults of the different species as follows: plates were washed with 3.5 ml water and incubated with 1.5 ml 5% bleach mixed 1∶1 with 5M NaOH for 10 min at room temperature. The eggs were washed with water and counted. A volume corresponding to 100–200 eggs, depending on the species, was added to each well of the drug plates. Plates were incubated at 20°C and scored microscopically for adults after 3 days and inspected again after 6 days for potential F2 generation of L_1_-larvae.

For the *S. ratti in vitro* tests 25 L3 larvae were incubated in 96-well plates containing 30 µl PBS buffer supplemented with 100 U/ml penicillin and 100 µg/ml streptomycin (Invitrogen) and appropriate drug dilutions. Control wells contained the highest percentage of solvent (2% DMSO). At each examination point (24, 48 and 72 h post-incubation) 15–20 µl of hot water (80°C) was added to each well, the larval movement observed and live worms counted using a dissecting microscope. All water stimulated wells were excluded from further reading. Half maximal effective concentration (EC_50_) values were calculated by non-linear regression of the sensitivity data, expressed as the percentage of surviving worms/larvae compared to the untreated control, to a sigmoidal dose-response curve of variable slope using Prism (GraphPad Prism version 5.00 for Mac OS X, GraphPad Software, San Diego California USA, http://www.graphpad.com).

### Discovery of new *Haemonchus contortus* DEG-3 subfamily members

A previously described procedure was followed for RNA extraction, cDNA synthesis and rapid amplification of cDNA ends by PCR (RACE-PCR) for *H. contortus* [10]. Briefly, total RNA was extracted from a pool of approximately 50 adult nematodes. To generate cDNA, 1 µg of total RNA was reverse transcribed to cDNA using a d(T)_30_ primer and a SuperScript II Reverse Transcriptase (Invitrogen). For RACE-PCR, an internal reverse primer (Supplementary Table S1) was combined with splice leader sequence (1 or 2) to obtain the 5′ untranslated region (UTR), or an internal forward primer combined with a poly-dT primer for the 3′ UTR of the transcript. We cloned and sequenced the full-length *Hco-acr-5*, *Hco-acr-17* and *Hco-acr-24* coding sequence from *H. contortus* cDNA (GenBank accessions GU109271-GU109279) using primer pairs NheI_acr-5_frw2.1 and NotI_acr-5_rev2.1, NheI_acr-24_frw1 and XhoI_acr-24_rev1, NheI_acr-17_frw1 and XhoI_acr-17_rev1 (Supplementary Table S1). PCR products were gel purified using the Wizard SV PCR Clean-Up kit (Promega) and cloned into pCRII-TOPO (Invitrogen). Plasmid DNA was purified using the QIAprep Spin Miniprep kit (Qiagen) and three clones of each gene were sequenced using the standard primers M13 forward and reverse. The reported sequences in Supplementary Figures S1, S2 and S3 are each from one of the nearly identical single clones.

### Accession numbers

*Hco-acr-5*, *Hco-acr-17* and *Hco-acr-24* coding sequence from *H. contortus* cDNA have been deposited with GenBank accessions GU109271-GU109279. MPTL-1 ACO48330 (GenBank).

SwissProt entries for mentioned proteins: ACC-1 Q21005_CAEEL, ACR-16 ACH1_CAEEL, ACR-17 P91320_CAEEL, ACR-20 B1Q281_CAEEL, ACR-21 Q9N5U8_CAEEL, ACR-23 O61884_CAEEL, ACR-5 ACR5_CAEEL, ACR-8 Q23355_CAEEL, AVR-14 Q95Q96_CAEEL, AVR-15 Q95PJ6_CAEEL, CUP-4 CUP4_CAEEL, DEG-3 ACH3_CAEEL, DES-2 ACH4_CAEEL, EXP-1 Q9TZI5_CAEEL, GAB-1 GBRB_CAEEL, GGR-2 Q2WF64_CAEEL, GLC-1 O17793_CAEEL, LEV-1 ACH7_CAEEL, LEV-8 Q93329_CAEEL, MOD-1 Q58AT9_CAEEL, PBO-5 Q67X94_CAEEL, UNC-29 ACH2_CAEEL, UNC-38 ACH5_CAEEL, UNC-49 Q0PDK2_CAEEL, UNC-63 ACH6_CAEEL, ZACN ZACN_HUMAN.

## Results/Discussion

### A phylogenomic pipeline

*Caenorhabditis elegans* peptide sequences annotated with the Gene Ontology term GO:0005230, ‘extracellular ligand-gated ion channel activity’, were retrieved from WormBase. These together with a similarly extracted set of human genes from uniprot and six *H. contortus* LGICs of the DEG-3 subfamily ([10] and Supplementary Figure S1, S2 and S3) were used as seed sequences for a Blast search against contiguous sequences from the abundant nematode, vertebrate and insect genome projects (Table 1). *Caenorhabditis elegans* is arguably the only finished eukaryote genome, but the genomes published as drafts are essentially complete, and several of the ongoing projects are well underway in terms of sequencing and assembly, only so far lacking in gene annotation. In this survey, we included data from 10 more nematode genomes, ranging from early shotgun stages to mature assemblies in annotation. Gene finding and annotation has become a major bottleneck, after next generation sequencing techniques accelerated sequence generation. By using Genewise to search the genome sequences directly we could also make use of unannotated genes. To assess nematode specificity of the herein predicted LGIC genes and to obtain more phylogenetic information, we also included three platyhelminth projects, four insect and nine vertebrate genomes (Table 1). No LGIC_LBD (from ligand binding domain, LBD) was found in eight plant species searched (www.gramene.org), which is in agreement with previous efforts [30]. The closest LGIC relatives in plants are highly diverged glutamate receptors [31]. Many plant toxins act on animal LGICs (e.g. curare, extracted from the plant *Strychnos toxifera* [32]). Due to the lack of LGICs, the toxic compounds pose little risk to the plants themselves.

### Method recall ability

The automated approach identified 84 out of 102 annotated *C. elegans* LGICs using the LGIC_LBD profile alone. Only one additional LGIC was identified when the 39 membrane binding domain hits, from the LGIC_MEMB profile, were also included. The recall of the profile itself from full-length peptides was nearly complete. Using hmmer2, all 102 were found with the LBD profile and 98 with the MEMB profile. The lower complexity of the *trans*-membrane domains and a presumed lower need for conserved sequence specificity, together with the often extensive and variable internal loop between *trans*-membrane domain 3 (TMD3) and TMD4, all complicated by a slightly larger number of introns, apparently makes the LGIC_MEMB Hidden Markov Model profile less successful for finding family members directly from genomic nucleotide data.

A domain centric approach, as used here, is highly useful to compare the whole spectrum of LGICs. The domain approach is straightforward, can be applied directly on sequence data without prior exon prediction and gives alignments where the aligned positions are largely comparable. It would also be much more challenging to align the protein family meaningfully over the full length. Inclusion of more variable regions e.g. the internal loop between TMD3 and TMD4 would make the interpretation more difficult. While the recall of *C. elegans* receptor subunit genes by the identification of the LBD domain directly from the genome is not complete (80%), it is reassuring that the full-length peptide results for genomes, where such are available, are similar to the ones obtained through searches on the genomic DNA level, in particular in the DEG-3 subfamily.

### Effects of genome project completeness

If genome sequence coverage is lacking altogether or if other problems keep the assembled contig size small, the number of LGICs predicted from our pipeline will be low. If the contigs with LGIC genes are too short so that they do not encompass the introns and exons for the LBD, they will not be detected by Genewise with a global (ls) type PFAM LGIC_LBD motif, even if fragments were detected by the initial BLAST screening.

To help assess the reliability of the number of genes found in the face of incompleteness of the ongoing projects, we measured the average contiguous sequence length (Table 1). Such a central measure can however be somewhat misleading for mature projects with a very high contig size variance. Indeed, the *B. malayi* and *L. scapularis* genome sequences show low sequence unit average length (<5 kbp), although the longest few contiguous sequences have considerable size (≫100 kbp; marked in Table 1). Gene counts for the genomes with average contig sizes below 3 kbp (*Ascaris suum*, *Nippostrongylus brasiliensis* and *Teladorsagia circumcincta*) in particular should not be taken for final.

### Additional DEG-3 homologs

**Figure 2:**
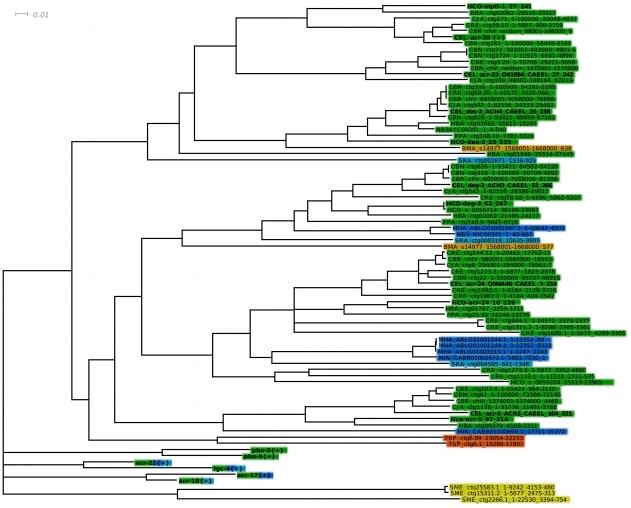
Detailed view of the DEG-3 sub-family. Detailed view of the DEG-3 sub-family from the LBD region NJ tree, with branches below 50% support after 1000 bootstrap iterations joined. A few related subfamilies are shown as collapsed branches. CEL, *Caenorhabditis elegans*, CBN, *C. brenneri*, CBR, *C. briggsae*, CRE, *C. remanei*, CJA, *C. japonica*, PPA, *Pristionchus pacificus*, HCO, *Haemonchus contortus*, HBA, *Heterorhabditis bacteriophora*, MHA, *Meloidogyne hapla*, NM3MIC/MIN, *M. incognitia*, NB3AYC *Ancylostoma ceylanicum*, SRA, *Strongyloides ratti*, BMA, *Brugia malayi*, TSP, *Trichinella spiralis*, SME, *Schmidtea mediterranea*. *Pristionchus pacificus* lacks a close MPTL-1 homolog and was predicted to be less sensitive to AAD-1566 than species such as *C. japonica*, *H. contortus* and *H. bacteriophora*.

The full length coding sequences of *H. contortus* genes *Hc-acr-17 (1590 bp)*, *Hc-acr-5 (1833 bp)* and *Hc-acr-24 (1698 bp)* were cloned by RACE PCR (see Materials and methods) and sequenced (Supplementary Figures S1, S2 and S3), helping to complete the understanding of individual DEG-3 subfamily members roles in monepantel drug action (Figure 2 and Table 2). Both *Hco-acr-5* and *Hco-acr-24* carried a spliced leader 2 (SL2) sequence at their 5′ end while *Hco-acr-17* had a spliced leader 1 (SL1) sequence. The predicted LGIC proteins possess motifs typical for Cys-loop ligand-gated ion channels, including an N-terminal signal peptide, with the exception of *Hco-acr-24* (as determined with Phobius [33], [34]), four transmembrane domains and the Cys-loop (two cysteines separated by 13 amino acids). Loops A to F, which are involved in ligand binding [35] are also present in the proteins. These loops are not annotated for *Hco-acr-17* as the alignment with other related nAChRs were the loops location are known is poor. In loop C, there are two adjacent cysteines, defining *Hco-acr-5*, *Hco-acr-17* and *Hco-acr-24* as nAChR α subunits. *Hco-acr-5* and *Hco*-acr-24 have the characteristic FxCC pattern, conserved among other ACR-5 and ACR-24 homologs, in contrast to *Hco*-acr-17 bearing the most common YxCC α subunit signature in loop C.

**Table 2:** Presence (+) or absence (−) of *C. elegans* homologous proteins of the DEG-3 subfamily members in various species.

Isolates	ACR-23	MPTL-1	ACR-20	DES-2	DEG-3	ACR-5	AAD-1566 EC_50_ in µM
*C. elegans*	+	−	+	+	+	+	0.19±0.05
*C. japonica*	+	−	+	+	+	+	<0.1
*C. briggsae*	+	−	+	+	+	+	0.90±0.06
*C. remanei*	+	−	+	+	+	+	1.25±0.29
*C. brenneri*	+^a^	−	+	+^a^	+^a^	+	0.38±0.08
*P. pacificus*	−	−	−	+	+	−	43±28
*C. elegans cb27*	−	−	+	+	+	+	25±16
*H. contortus* b	−	+	−	+	+	+	0.003
*H. bacteriophora*	−	+	−	+	+	+	n. d.
*B. malayi* b	−	−	−	+	+	−	n. d.
*S. ratti* b	−*	−*	−*	−*	+	−	>250
*M. incognita* b	−	−	−	−	+	+	n. d.
*M. hapla* b	−	−	−	−	+	−	n. d.
*T. spiralis* b	−	−	−	−	−*	−*	n. d.
Vertebrates	−	−	−	−	−	−	n. d.

The AAD-1566 EC_50_ values with standard errors of the mean was calculated using sigmoidal dose-response (variable slope) curves from *in vitro* data points with four replicates. *Haemonchus contortus* EC_50_ was determined in a previous study [10].

a Multiple copies of the gene are found in the genome.

b Obligatory parasites.

c See Table 1 for a list of vertebrate species analysed.

*: *S. ratti* has a more remote relative of MPTL-1/DES-2 kind, much as *T. spiralis* of DEG-3/ACR-5.

### Detecting LGICs

We used our phylogenomic pipeline on 33 genomes of varying levels of completeness, detecting 1273 putative genes bearing the PFAM LGIC_LBD motif (Table 1). The average number found in nematode genomes with an average sequence unit larger than 3 kbp was 56.1, whereas the same number was 41.7 for vertebrates, 18.0 for insects and 0 for plants. We also searched the nembase3 and nematode.org expressed sequence tag sets, finding a total of 27 LGICs with the LBD motif. An average of 31 and 57 LGICs were found in parasitic and non-parasitic organisms, respectively. The trend among the nematodes is clearly in agreement with the hypothesis that parasites have a reduced number of LGICs. It has been suggested that this could be a consequence of the less variable environment they encounter in comparison with their free-living relatives [19].

There is also considerable variation in LGIC number among the vertebrates (Table 1). The teleost genomes show a larger set of LGIC, in comparison to, for example, *Bos taurus* and *Homo sapiens*. The teleost repertoire appears to consist largely of multiple closely related variants of the terrestrial vertebrate LGIC types. The nematodes show a larger repertoire (Figure 1).

While the platyhelminthes included in the survey showed a smaller overall number of LGICs, they did have several unique types. LGIC subunits that are known to constitute part of drug target receptors are labelled in Figure 1. It is interesting to see how these drug target subunit genes form rather broad, i.e. member rich, yet nematode specific sub-branches of the superfamily tree. Importantly, the DEG-3 family appears nematode specific. In an optimistic outlook, several other such broad nematode specific branches exist in the tree, which could potentially be exploited as new anthelmintic targets.

Co-segregation with known named seed sequences in the bootstrapped tree was used for assigning putative identity to homologous genes. Interestingly, we found that neither *P. pacificus* nor *S. ratti* carries an ortholog of *Hco-mptl-1* (Figure 2). Based on a single drug target model we thus predict *P. pacificus* and *S. ratti* to be insensitive to monepantel. We proceeded to test this hypothesis *in vitro*.

### *In vitro* assay

**Figure 3:**
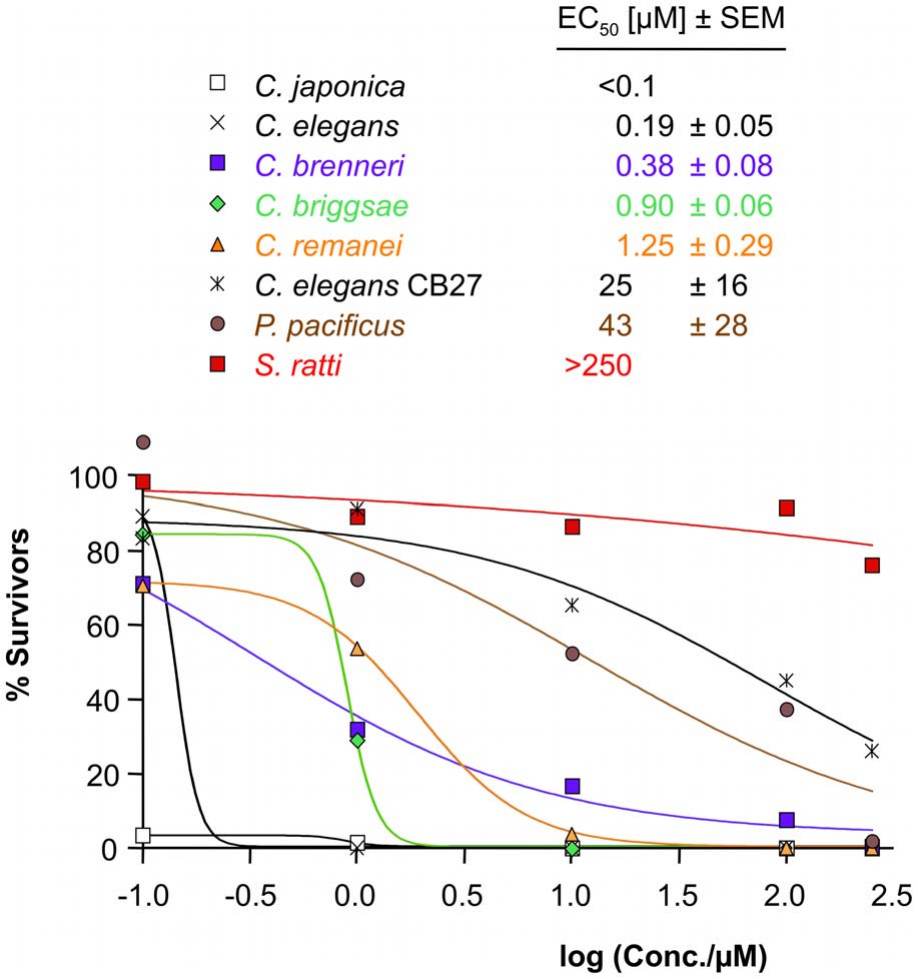
Sensitivity to AAD-1566 determined *in vitro*. The percentage of the average number of adult worms present after 3 days exposure relative to a control is plotted versus drug concentration for *Caenorhabditis elegans*, mutated *C. elegans* strain *acr-23 (cb27)*, *C. japonica*, *C. briggsae*, *C. remanei*, *C. brenneri*, *Pristionchus pacificus* and *S. ratti*. Sigmoid dose-response curve fit was performed in Prism. EC_50_ values with standard errors, estimated from data points with four replicates, are shown inset.

An *in vitro* assay was established. Nematodes of one species were grown on 24 well NGM plates where each four well column was treated with a different drug concentration. An equal amount of eggs was added to each well, and the nematodes were scored microscopically after 3 and 6 days. A final concentration of 1% DMSO was used in all wells for the drug tests, including the no-drug controls (Figure 3, Supplementary Figure S4). All species tested (*C. elegans*, *C. japonica*, *C. briggsae*, *C. brenneri*, *C. remanei*, *P. pacificus*) tolerated up to 1% DMSO (Supplementary Figure S4 and Supplementary Table S2). For *S. ratti* the highest percentage of solvent (2% DMSO) found in the plate was used in the control wells and well tolerated.

All Clade V species tested (*C. elegans*, *C. japonica*, *C. briggsae*, *C. brenneri*, *C. remanei*, *P. pacificus*) exhibited a similar sensitivity to ivermectin (EC_50_≥10 nM; Supplementary Figure S7 and Supplementary Table S3). *S. ratti* L3 exposed to ivermectin at concentrations of 10 µM and above showed decreased survival rates 24–72 h post-incubation (EC_50_ of 13.6 µM 72 h post-incubation) (Supplementary Figure S7). This served as an additional positive control for the methods. It appears likely that drug sensitivity can be consistently determined for all strains. In a similar experiment the assayed nematode species showed varying degrees of concentration-dependent sensitivity towards DMSO, used as a solvent for the drugs. Even a compound with a small effect in this *in vitro* test could still be of therapeutic value, as levamisole clearly demonstrates. Levamisole does not directly kill the parasitic nematodes but creates a short term reversible paralysis, sufficient to allow the host to e.g. expulse the worms [12]. Up to mmolar concentrations of levamisole did not produce any effect detectable by our test readout (data not shown).

### *Hco-mptl-1* ortholog detection predicts AAD-1566 sensitivity - an MPTL-1/ACR-23 ortholog is the primary target

The present study tests the hypothesis that MPTL-1 is a major target of monepantel, since a nematocide effect on *P. pacificus* or *S. ratti*, which lacks an MPTL-1 homolog, would negate this. The *P. pacificus* genome has been published in a draft state, and as the assembly is nearly complete it is unlikely, but not impossible, that an eventual *acr-23* ortholog could have been missed. The *S. ratti* genome is still in progress, but we were able to detect a subunit that appears branched prior to the ACR-23/MPTL-1/ACR-20 and the DES-2 split (see Figure 2 and Table 2). This species arguably helps us narrow down how deep the sensitivity to monepantel reaches in the tree.

Among the species *C. elegans*, *C. japonica*, *C. briggsae*, *C. brenneri*, *C. remanei*, *P. pacificus* as well as the mutated *C. elegans* strain *acr-23 (cb27)*, *C. japonica* was the most sensitive to monepantel with EC_50_ values in the low nM range (Figure 3 and Table 1). This is comparable to the results previously obtained for *H. contortus* [10]. *C. elegans* was strongly affected at 1 µM, with an estimated EC_50_ of 0.19 µM. *C. remanei*, *C. briggsae* and *C. brenneri* showed similar EC_50_ values, but we found a comparatively large number of adult *C. brenneri* even at higher µM concentrations, e.g. 7.6% at 100 µM (Supplementary Table S4). *C. brenneri* has the largest assembly of LGICs in the study, and also possesses an extra DES-2 paralog and an additional ACR-23. Closer examination of the sequences of these copies did not present a convincing explanation of the diminished phenotype. One explanation may possibly lie in the gene doses of the channel subunits, leading to different stochiometries of the assembled channels, as has been observed *in vitro* [36].

The difference in EC_50_ value between *C. japonica* and to the other sensitive worms in the Caenorhabditis genera is already large. While we would not venture a molecular correlate, it is interesting to observe that the both the Cjp-ACR-23 and Cjp-ACR-20 seem to have diverged somewhat from the other sensitive Caenorhabditis worms, branching prior to them, possibly retaining more of an element important for high sensitivity, common with the earlier branched *Hco*-MPTL-1.

*Pristionchus pacificus* is rather insensitive to monepantel with an EC_50_ of 43 µM (Table 2, Figure 3). Furthermore, our *in vitro* test with *S. ratti*, bearing an early branching relative of ACR-20/ACR-23/MPTL-1, showed that monepantel lacks activity against *S. ratti*. A survival rate of 69% was observed after 72 h even with the highest concentration (250 µM) tested (Figure 3). A direct molecular mechanism is beyond the scope of the present investigation. However, we found that the phylogenomic detection of the ortholog of *Hco-mptl-1*, previously found mutated in strains insensitive to AAD-1566 [10], coincides with sensitivity to AAD-1566. This in agreement with our hypothesis that MPTL-1 is a major target of the drug.

### *Caenorhabditis elegans* mutated in the DEG-3 family

*acr-23 (cb27)*, a strain of *C. elegans* exhibiting a large deletion in *Cel-acr-*2*3* [8], was much less sensitive than wild type (genome strain N2), with an EC_50_ of 25 µM (Table 2, Figure 3). The difference in growth was marked and clearly visible to the naked eye (Supplementary Figure S5). This test can naturally not rule out the involvement of other LGIC subunits or indeed other genes in the susceptibility to AAD-1566. However, a set of *C. elegans* strains mutant only in other genes of the DEG-3 family (DEG-3/DES-2, ACR-5, ACR-18, and ACR-20) showed no loss of sensitivity towards AAD-1566 (Supplementary Figure S6 and Supplementary Table S5). This further strengthens the hypothesis that a subunit orthologous to MPTL-1/ACR-23 is required for the observed effect.

### Detailed phenotype suggests additional secondary target – sensitivity stays in the family

For species that possess an MPTL-1 ortholog (e.g. *C. elegans* with Cel-ACR-23), AAD-1566 is lethal in vitro at nM concentrations, and a concentration-dependent retardation of development was observed. The strains without an *Hco*-MPTL-1 ortholog (*P. pacificus* and *C. elegans acr-23 (cb27)*) also exhibited a drug concentration-dependent developmental retardation. However, the substance was not lethal to them at the tested concentrations, as growth could still be observed after 6 days. Also in the case of *S. ratti* the survival rate of the larvae was slightly affected at high drug concentration (69% at 250 µM) and less at lower concentration. This suggests that there is at least one additional target.

One candidate is DES-2. In nematode strains selected for loss of sensitivity to AAD-1566, mutations in addition to those affecting *Hco-mptl-1* were found in the *Hco-des-2* gene 5′ UTR, introducing two novel upstream open reading frames, possibly reducing protein expression [10]. All tested species possess the DES-2 ortholog that bears the highest similarity to the established target outside the *Cel*-ACR-20/*Cel*-ACR-23 branch. If MPTL-1 is a primary target, causing high nematode lethality from AAD-1566, strains with modulations in the expression of a second target, DES-2, would only be selected for once MPTL-1 sensitivity was lost. It was noted in proof that in a recent study [37] Rufener et al. have expressed a functional *H. contortus* DES-2/DEG-3 channel in *Xenopus* oocytes that shows monepantel sensitivity. Though active against a range of clade V gastrointestinal nematodes, monepantel was reported to have only limited efficacy against *Trichuris ovis* (clade I) [38]. Genomic information to correlate this result with the absence of MPTL-1/ACR-23/DES-2 homologous subunits would be interesting.

There are a number of nematodes that, based on their complement of predicted nAChR genes, would be interesting to test for their sensitivity to AADs, but this would require other test methods. Two *Meloidogyne* species bear no close MPTL-1 homologs but have an ACR-5, homolog, which *P. pacificus* lacks. *Heterorhabditis bacteriophora* carries a DEG-3 family complement, which is highly similar to *H. contortus*, and we would thus predict a similar drug effect. Some important human parasitic nematodes of the clade I (*Trichinella spiralis*) and III (*Brugia malayi*) have more distant DES-2/DEG-3 homologs, much like *Schmidtea mediteranea*. A conjecture would be that they would show sensitivity only at a higher concentration. Tests on them could perhaps show what level of sequence identity is required, or what regions of the subunit need to be conserved, for any paralysis effect to be seen.

### Conclusions

The family of LGIC provides many important drug and toxin targets, with nematodes bearing several unique subfamilies well diverged from those of other eukaryotes. We have constructed a simple phylogenomic pipeline to detect LGIC subunit genes. We survey the gene family in the many complete and ongoing sequencing projects in the nematode phylum and contrast these to genomes from some other relevant phyla to establish that the DEG-3 family indeed appears nematode specific to date. The survey also establishes the detection of drug sensitivity groups.

Given the hypothesis that an MPTL-1 homolog is the primary target of monepantel, the phylogenomic information gathered predicts *P. pacificus* and *S. ratti* to be insensitive to the drug, while four other model nematode species were predicted to be sensitive. These conjectures were tested experimentally. The *in vitro* effect of AAD-1566 on the panel of nematodes was found consistent with the hypothesis. All data point towards MPTL-1 as a primary target, in agreement with previous studies. We further hypothesise an additional secondary target for AAD-1566, possibly DES-2. This would explain a dose dependent growth retardation effect that is largely masked by the stronger, MPTL-1 mediated response.

## Supporting Information

Figure S1 *Haemonchus contortus Hco-acr-5* cDNA sequence. Nucleotide sequence from *Haemonchus contortus Hco-acr-5* cDNA with conceptual peptide translation. Putative transmembrane domains TMD1-TMD4 are highlighted in grey. Prominent conserved LGIC α subunit loops are highlighted in green. Note the C-loop motif FxCC, typical for ACR-5. Export signals, as predicted by Phobius, are highlighted blue.
(0.09 MB PDF)

Figure S2 *Haemonchus contortus Hco-acr-17* cDNA sequence. Nucleotide sequence from *Haemonchus contortus Hco-acr-17* cDNA with conceptual peptide translation. Putative transmembrane domains TMD1-TMD4 are highlighted in grey. Prominent conserved LGIC α subunit loops are highlighted in green. Export signals, as predicted by Phobius, are highlighted blue.
(0.46 MB PDF)

Figure S3 *Haemonchus contortus Hco-acr-24* cDNA sequence. Nucleotide sequence from *Haemonchus contortus Hco-acr-24* cDNA with conceptual peptide translation. Putative transmembrane domains TMD1-TMD4 are highlighted in grey. Prominent conserved LGIC α subunit loops are highlighted in green. Export signals, as predicted by Phobius, are highlighted blue.
(0.88 MB PDF)

Figure S4 *In vitro* test of DMSO tolerance. Live adults on NGM wells after 3 days from egg deposition, in percent compared to control (0% DMSO) average, was plotted against DMSO concentration for *Caenorhabditis elegans*, *C. japonica*, *C. briggsae*, *C. remanei*, *C. brenneri* and *Pristionchus pacificus*. Green field background denotes presence of progeny after 6 days, indicating the ability to complete a whole life cycle. Error bars represent the SEM from three replicates. All species tolerated the typical dose of 1% DMSO. 2.5% leads to a marked development retardation.
(0.29 MB PDF)

Figure S5 Comparison of phenotype between Cel N2 (wild type) and acr-23 (cb27) after AAD-1566 exposure. N2 worms were more sensitive, with the bacterial lawn intact down to drug doses of 1 µM, whereas feeding activity was noticeable up to 100 µM, with 1 µM being visually no more affected than the control after 6 days.
(3.32 MB PDF)

Figure S6 Sensitivity to AAD-1566 determined *in vitro*. The percentage of the average number of adult worms present after 3 days exposure relative to a control is plotted versus drug concentration for *Ceanorhabditis elegans*, and mutant strains VC1598, TU1803, NC293 and RB1226. Error bars represent the SEM from four replicates. Sigmoid dose-response curve fit was performed in Prism.
(0.13 MB PDF)

Figure S7 Sensitivity to ivermectin determined *in vitro*. The percentage of the average number of adult worms present after 3 days exposure relative to a control is plotted versus drug concentration for *Caenorhabditis elegans*, *C. japonica*, *C. briggsae*, *C. remanei*, *C. brenneri* and *Pristionchus pacificus*. Sigmoid dose-response curve fit was performed in Prism. EC_50_ values with standard errors, estimated from data points with four replicates, are shown inset.
(0.13 MB PDF)

Table S1 Primers used for PCR amplification. Primers used for PCR amplification of 18s rRNA of *Caenorhabditis sp.* or *Pristionchus pacificus* and *deg-3* subfamily genes from *Haemonchus contortus*.
(0.06 MB PDF)

Table S2 Sensitivity to DMSO determined *in vitro*. Number of adult worms present after 3 days exposure for *C. elegans*, *C. japonica*, *C. briggsae*, *C. remanei*, *C. brenneri* and *P. pacificus*. Green field background denotes presence of progeny after 6 days, indicating the ability to complete a whole life cycle. Yellow fields in t-test rows indicate that the hypothesis of the counts of that concentration being drawn from a normal distribution with the same average as the control (0%) could not be rejected at a 95% confidence level (two-tailed heteroscedastic t-test).
(0.06 MB PDF)

Table S3 Sensitivity to ivermectin determined *in vitro*. Number of adult worms present after 3 days exposure for *C. elegans*, *C. japonica*, *C. briggsae*, *C. remanei*, *C. brenneri* and *P. pacificus*. Yellow fields in t-test rows indicate that the hypothesis of the counts of that concentration being drawn from a normal distribution with the same average as the control (0%) could not be rejected at a 95% confidence level (two-tailed heteroscedastic t-test).
(0.06 MB PDF)

Table S4 Sensitivity to AAD-1566 determined *in vitro*. Number of adult worms present after 3 days exposure for *C. elegans*, *C. japonica*, *C. briggsae*, *C. remanei*, *C. brenneri* and *P. pacificus*. Green field background denotes presence of progeny after 6 days, indicating the ability to complete a whole life cycle. Yellow fields in t-test rows indicate that the hypothesis of the counts of that concentration being drawn from a normal distribution with the same average as the control (0%) could not be rejected at a 95% confidence level (two-tailed heteroscedastic t-test).
(0.06 MB PDF)

Table S5 Sensitivity to AAD-1566 determined *in vitro* for mutant *C. elegans* isolates VC1598: *Cel-acr-20(ok1849)/mT1 II*; *+/mT1[dpy-10(e128)] III*, NC293: *Cel-acr-5(ok180) III*, TU1803: *Cel-deg-3(u662) Cel-des-2(u695) V* and RB1226: *Cel-acr-18(ok1285) V*. Green field background denotes presence of progeny after 6 days, indicating the ability to complete a whole life cycle. Yellow fields in t-test rows indicate that the hypothesis of the counts of that concentration being drawn from a normal distribution with the same average as the control (0%) could not be rejected at a 95% confidence level (two-tailed heteroscedastic t-test).
(0.05 MB PDF)
