# Supplementary material for: Phylogenomics of Ligand-Gated Ion Channels Predicts Monepantel Effect
Source: PLoS Pathog. 2010 Sep 9;6(9):e1001091. doi: 10.1371/journal.ppat.1001091 (PMC2936538; doi:10.1371/journal.ppat.1001091)
Supplement: Figure S1 — Haemonchus contortus Hco-acr-5 cDNA sequence. Nucleotide sequence from Haemonchus contortus Hco-acr-5 cDNA with conceptual peptide translation. Putative transmembrane domains TMD1-TMD4 are highlighted in grey. Prominent conserved LGIC α subunit loops are highlighted in green. Note the C-loop motif FxCC, typical for ACR-5. Export signals, as predicted by Phobius, are highlighted blue. (0.09 MB PDF) [file ppat.1001091.s001.pdf]

Supplementary Figure 1

Hco-acr-5H

```
1    ATGGTTA TCATAA TTCTGT TGACG CTGAC ATACAC GGAATG TTTAGA GAAAT TCGGA AAACAA ATTAGC TTGTC GACGTC CACCT ATAGCT ACAAGA GAT  100
1    M  V  I  I  I  L  L  T  L  T  Y  T  E  C  L  E  K  F  G  K  Q  I  S  L  S  T  S  T  Y  S  Y  K  R  W  34
     Export signal
101  GGTATGA CGAGAA TCGGCA ACATA GCTCT GAGCTC AACAAC GGACTT CAAGA AGAAT ATCACA TGCACT TGCGA AGAAGC TTGGA AAATGG TAATGC GAC  200
35    Y  D  E  N  R  Q  H  S  S  E  L  N  N  G  L  Q  E  E  Y  H  M  H  L  R  R  S  L  E  N  G  N  A  T  67

201  GACTTTA AATCGG ACATTT CTTGC TCAGC TGGCGG AGGAGC GTCGGA AGTTC TTTGA GAACCT GAATGC TGGTG CATTCA TGAAC GACCAA CGTCGA CTG  300
68   T  L  N  R  T  F  L  A  Q  L  A  E  E  R  R  K  F  F  E  N  L  N  A  G  A  F  M  N  D  Q  R  R  L  100

301  GTGGACG ATTTGC TAGACG AGAGC TACTA CGAGAA ACACGT CCATCC TCGGG TAGAT CACCTG AAGCCA ACTCG GATCAA CGTCA GCATGA GTCTTT ATC  400
101  V  D  D  L  L  D  E  S  Y  Y  E  K  H  V  H  P  R  V  D  H  L  K  P  T  R  I  N  V  S  M  S  L  Y  Q  134

401  AAATCCT CGACGT GGACGA ACACT CGCAA AGTATC GTGGTA AACGTG TGGAT GGTGC AGAATT GGTACG ATGAA TTTCTT GACTG GGATCC CAGAGA GTA  500
135   I  L  D  V  D  E  H  S  Q  S  I  V  V  N  V  W  M  V  Q  N  W  Y  D  E  F  L  D  W  D  P  R  E  Y  167
                                                       D-loop
501  TGGGATG TTGAAC AAAACC ATAGT ACCCT ATGATC AGATTT GGATTC CGGAT ACCTA CTTGTA CAACAG TGAAG CGTTGG AACGA AAGAAA ACGAAA TCG  600
168  G  M  L  N  K  T  I  V  P  Y  D  Q  I  W  I  P  D  T  Y  L  Y  N  S  E  A  L  E  R  K  K  T  K  S  200
                                            A-loop
601  ATAATGA ATGCGA TAGTGG AGACG GGATT TTGGTC GAACAA CAGCCT TGGGG CCAGA GTTCAG CTTATG TTTCC GGCTAT TTATA AGTTGT CTTGCG CCA  700
201  I  M  N  A  I  V  E  T  G  F  W  S  N  N  S  L  G  A  R  V  Q  L  M  F  P  A  I  Y  K  L  S  C  A  M  234
     E-loop
701  TGAACGT GCAATG GTTTCC GTACG ACTCG CAGAAC TGTACA TTCATC ATTTC GAGTT GGACCC ATGATA AGGCT ACGATC GATTA CTGGGC CGAGCA CCC  800
235   N  V  Q  W  F  P  Y  D  S  Q  N  C  T  F  I  I  S  S  W  T  H  D  K  A  T  I  D  Y  W  A  E  H  P  267
     Cys-loop                                            B-loop
801  AACTGTG AATTTG AAGAAT ATGGC CCGAA ACGATG AATGGG AAGTGC TCGGA TTTGA GTTCGA ACGTAT AGAGC AAATCT TCAAA TGTTGT GCAAAT CCG  900
268   T  V  N  L  K  N  M  A  R  N  D  E  W  E  V  L  G  F  E  F  E  R  I  E  Q  I  F  K  C  C  A  N  P  300
     F-loop                                                C-loop
901  TGGGTAC TGCTCT ACGCAC ATTTG GTGAT TCGAAG AAAGCC GCTTTA CTATG TTATC AACCTT GTAATC CCTAC GTCAAT CATCA CAATCG TGGCTA TCA  1000
301  W  V  L  L  Y  A  H  L  V  I  R  R  K  P  L  Y  Y  V  I  N  L  V  I  P  T  S  I  I  T  I  V  A  I  T  334
                                            TMD1
1001 CTGGTTT CTTCAC ACCCAC TTCAA CCTCC AGTGAA CGTGAT GAAAAA CTGTA TCTCG GTATCA ACACAC TTCTC ACAATG TCAAT TATGAT GCTTAT GGT  1100
335   G  F  F  T  P  T  S  T  S  S  E  R  D  E  K  L  Y  L  G  I  N  T  L  L  T  M  S  I  M  M  L  M  V  367
                                                TMD2
1101 ATGCAAC CAGATG CCGTCA ACTTC GTCGT ATGTAC CACTGA TGAGTT GGTAC TACCT TGGAAT CATCAT CGTCA TTGTGC TCGGA ACCCTA CTGGCT ACG  1200
368   C  N  Q  M  P  S  T  S  S  Y  V  P  L  M  S  W  Y  Y  L  G  I  I  I  V  I  V  L  G  T  L  L  A  T  400
                                                 TMD3
1201 ATAGTTC TAGCGA TTCACG GTCAA AAGCG TCACAA TCGACC ATTGTC ACGAT GGGCA CGTGGT CTCGTG CTTAA CCGCTT CGTTG AGACCT TCATAC TGA  1300
401  I  V  L  A  I  H  G  Q  K  R  H  N  R  P  L  S  R  W  A  R  G  L  V  L  N  R  F  V  E  T  F  I  L  R  434

1301 GACCACC GTGTGC ACTCGT CGACT TGTGG AGCGAA TACGGT ATCGTC GAAGA ACGTC GTCTGT CGATGT CGAAG CTTGAC CCACT TCTAGT GCAGCA ACT  1400
435   P  P  C  A  L  V  D  L  W  S  E  Y  G  I  V  E  E  R  R  L  S  M  S  K  L  D  P  L  L  V  Q  Q  L  467

1401 TGATCCT ATCTCT CTGATG CCTCC CCGAC CTCGAG CATTTT TCGGCT CCATT TCGTC GAACAT GTCGGA AACGT CGTCCT ACTCC TATACA ACAAGG CTT  1500
468  D  P  I  S  L  M  P  P  R  P  R  A  F  F  G  S  I  S  S  N  M  S  E  T  S  S  Y  S  Y  T  T  R  L  500

1501 GCGACTC TAACCC GACAGT ACACC ACTCA GGTACG AGTAAG AGAAAA AGAGA GGCAG TCCTTC CTCCTC AAGCA GTCTCA TCATG CACGAA GTGTTA AGC  1600
501  A  T  L  T  R  Q  Y  T  T  Q  V  R  V  R  E  K  E  R  Q  S  F  L  L  K  Q  S  H  H  A  R  S  V  K  R  534

1601 GACAAAA AATGAG CAGGAG GTGTG CTCTC GAATGG GAGTAC CTAGCA AATGT GATCG ATCGTG TACTGT TAACG GTGTTC TTCGT AGTGAC TATGTC CCT  1700
535   Q  K  M  S  R  R  C  A  L  E  W  E  Y  L  A  N  V  I  D  R  V  L  L  T  V  F  F  V  V  T  M  S  L  567
                                                                   TMD4
1701 TTTCTTG CTTCTG GTTGCT TTTGA CTATC TCTTTA CCGTAC ACAATC ATGTC CAGTAG  1758
568   F  L  L  L  V  A  F  D  Y  L  F  T  V  H  N  H  V  Q  *  585
```
