# Supplementary material for: Phylogenomics of Ligand-Gated Ion Channels Predicts Monepantel Effect
Source: PLoS Pathog. 2010 Sep 9;6(9):e1001091. doi: 10.1371/journal.ppat.1001091 (PMC2936538; doi:10.1371/journal.ppat.1001091)
Supplement: Figure S2 — Haemonchus contortus Hco-acr-17 cDNA sequence. Nucleotide sequence from Haemonchus contortus Hco-acr-17 cDNA with conceptual peptide translation. Putative transmembrane domains TMD1-TMD4 are highlighted in grey. Prominent conserved LGIC α subunit loops are highlighted in green. Export signals, as predicted by Phobius, are highlighted blue. (0.46 MB PDF) [file ppat.1001091.s002.pdf]

Supplementary Figure 2

Hco-acr-17H

```
     1 ATGATCAGGATGTTAGCAATTCTTACATTATACCCTATGGTCCGAGCCGAAGGCCAACAGCAACAGCTAATGGCCGAGTTATTCGGTAACTACAGGAAAG 100
     1 M  I  R  M  L  A  I  L  T  L  Y  P  M  V  R  A  E  G  Q  Q  Q  Q  L  M  A  E  L  F  G  N  Y  R  K  E 34
       Export signal
   101 AATTGAGACCAGTGAAATCGTTTGATTCGGGTCCCACAAATGTCACTGTTCAATTATACTTCAAGCAAATCCAAAAAGTACAAGAAAATGATCAGATAAT 200
    35  L  R  P  V  K  S  F  D  S  G  P  T  N  V  T  V  Q  L  Y  F  K  Q  I  Q  K  V  Q  E  N  D  Q  I  I  67

   201 TACCATCTACTGCTGGCTCGAAGAGTACTGGTATGACGAATTCCTTACGTGGAAACCTGAAGAATTTGGCGGCGTCAAGGAACTTCACGTGCCTTCACAA 300
    68  T  I  Y  C  W  L  E  E  Y  W  Y  D  E  F  L  T  W  K  P  E  E  F  G  G  V  K  E  L  H  V  P  S  Q   100

   301 ATGATTTGGCGACCAGATTTGCTAGTGTACAATAATGCCAACATGAACATACGAGAAAACGAGATGCAAACGAATGTCCAGATCGAACACACTGGCCGCA 400
   101 M  I  W  R  P  D  L  L  V  Y  N  N  A  N  M  N  I  R  E  N  E  M  Q  T  N  V  Q  I  E  H  T  G  R  I 134

   401 TTTCGCTATTCCGTGCCCTGATCACCGATATCACTTGCGATCTACGGCTTGAGCGATTTCCTTACGATCAGCAAATTTGTTACATTTTACTGGCGTCGTG 500
   135  S  L  F  R  A  L  I  T  D  I  T  C  D  L  R  L  E  R  F  P  Y  D  Q  Q  I  C  Y  I  L  L  A  S  W  167
                                        Cys-loop
   501 GTCGTATGACGGCTCACAAATCATGCTTTATACAGCGGAGGAGCCTACAGCTGAACCAAGCACGAACAGGCATTCTGCGCAACAAAATCCTCTCAGGACC 600
   168  S  Y  D  G  S  Q  I  M  L  Y  T  A  E  E  P  T  A  E  P  S  T  N  R  H  S  A  Q  Q  N  P  L  R  T   200

   601 AATCTCGCCGCACTAAATCACTATATTCCTAATATGGAATGGACACTGGTGGATTTCAAGTACAGGAGCAATCTCAAATATTACGATTGCTGTCCAAATC 700
   201 N  L  A  A  L  N  H  Y  I  P  N  M  E  W  T  L  V  D  F  K  Y  R  S  N  L  K  Y  Y  D  C  C  P  N  P 234

   701 CATATCCGGACATTTCGTACTTTTTTGCTATCAAACGGAACCCGTCATACTATCTATTTACTCTGATCATTCCGTCTGCGTTCATCACAATCGTTACGGT 800
   235  Y  P  D  I  S  Y  F  F  A  I  K  R  N  P  S  Y  Y  L  F  T  L  I  I  P  S  A  F  I  T  I  V  T  V  267
                                                   TMD1
   801 GATCGGTTTCTTCACGCCACATTCATCAACTGGTGAAAACACCGAGAAGGTCAGTCTGGGAGTCACTGCTCTGCTGTCTTTGGCTATAATACTGATGATG 900
   268  I  G  F  F  T  P  H  S  S  T  G  E  N  T  E  K  V  S  L  G  V  T  A  L  L  S  L  A  I  I  L  M  M  300
                                                     TMD2
   901 GTTTCCGACAAACTACCAGCCACGTCCAACTCCGTACCGTTGCTAGGGCAATACTATATTGGTCTGATATTCATCATGTTCCTCGCCACTTATTGCACGA 1000
   301 V  S  D  K  L  P  A  T  S  N  S  V  P  L  L  G  Q  Y  Y  I  G  L  I  F  I  M  F  L  A  T  Y  C  T  T 334
                                                      TMD3
  1001 CGTTCACGCTTGGGCTTCAGATGCAGGGAAATACGGGCCAACCTATTTCCCGTCGAGTACGTGGGTTCCTACTCTCAATACGAGTTAACAAATCTTCGTT 1100
   335  F  T  L  G  L  Q  M  Q  G  N  T  G  Q  P  I  S  R  R  V  R  G  F  L  L  S  I  R  V  N  K  S  S  L  367

  1101 ACTTCAATGGTTTTTCGGTAAAGAACTGATGAACACCCAGGAAACCATCAAGATGCGGTTAAAAAAATACGACAAGTTGTCCGAATTGAAGAAAACATTT 1200
   368  L  Q  W  F  F  G  K  E  L  M  N  T  Q  E  T  I  K  M  R  L  K  K  Y  D  K  L  S  E  L  K  K  T  F   400

  1201 GCCAAGAACTTCGTGACTTTCCAAACAAAGTTCCTCAAGACAGAAGAACCGAAGCATCAAAAGTTACCCATGATTGACAGCAATGAGGAAGAGGAGGCCA 1300
   401 A  K  N  F  V  T  F  Q  T  K  F  L  K  T  E  E  P  K  H  Q  K  L  P  M  I  D  S  N  E  E  E  E  A  M 434

  1301 TGCGTAAAGTAAACGATCCTATGCTGCATAACATAGTCATCGACCTTTTGGAAGGTGTCCAAGCTATCAGGCAAGAAATGCTTATGCAGGAGCATGTGAA 1400
   435  R  K  V  N  D  P  M  L  H  N  I  V  I  D  L  L  E  G  V  Q  A  I  R  Q  E  M  L  M  Q  E  H  V  K  467

  1401 ACGAATTCGAAGAGAATGGCAAATGTTGGCCAGAATGATGGAAAAGATGATCATGTGGGTATTCATTGTGTGTACGATTCTATTCGCCTTATTTATGCTC 1500
   468  R  I  R  R  E  W  Q  M  L  A  R  M  M  E  K  M  I  M  W  V  F  I  V  C  T  I  L  F  A  L  F  M  L  500
                                               TMD4
  1501 TACGATCGACAAGATCCGCCAGTGATCACTGATGAATACATGAAAGAGAAGGCAGACGCTTGA 1563
   501 Y  D  R  Q  D  P  P  V  I  T  D  E  Y  M  K  E  K  A  D  A  *   520
```
