# Supplementary material for: Phylogenomics of Ligand-Gated Ion Channels Predicts Monepantel Effect
Source: PLoS Pathog. 2010 Sep 9;6(9):e1001091. doi: 10.1371/journal.ppat.1001091 (PMC2936538; doi:10.1371/journal.ppat.1001091)
Supplement: Figure S3 — Haemonchus contortus Hco-acr-24 cDNA sequence. Nucleotide sequence from Haemonchus contortus Hco-acr-24 cDNA with conceptual peptide translation. Putative transmembrane domains TMD1-TMD4 are highlighted in grey. Prominent conserved LGIC α subunit loops are highlighted in green. Export signals, as predicted by Phobius, are highlighted blue. (0.88 MB PDF) [file ppat.1001091.s003.pdf]

Supplementary
Figure 3

Hco-acr-24H

```
         ---------|---------|---------|---------|---------|---------|---------|---------|---------|---------|
   1 ATGAATGAACGGGGTGAATTTCTTCCGCATATACGGTTGGCTAAAGATTTAATGGATAAACGAAGATATGACTCTCGAGTACGACCGGTCATGAATCATT 100
   1 M  N  E  R  G  E  F  L  P  H  I  R  L  A  K  D  L  M  D  K  R  R  Y  D  S  R  V  R  P  V  M  N  H  S 34

         ---------|---------|---------|---------|---------|---------|---------|---------|---------|---------|
 101 CACAACCAACTACCGTCGTGTTTTCGATGAGTCTCTATCAAATTTTGGCCATCAATGAAAAACGTCAAAGTGTCGATCTGAATGTATGGGTGATCCAAAA 200
  35  Q  P  T  T  V  V  F  S  M  S  L  Y  Q  I  L  A  I  N  E  K  R  Q  S  V  D  L  N  V  W  V  I  Q  K  67
                                                                                                D-loop
         ---------|---------|---------|---------|---------|---------|---------|---------|---------|---------|
 201 GTGGAAAGACGATTTCCTTGGTTGGAATCCATACCTATACGGTATGATAAACACCACGATACTACCGTATGAAGCAATCTGGTTACCGGATACGTACGTA 300
  68  W  K  D  D  F  L  G  W  N  P  Y  L  Y  G  M  I  N  T  T  I  L  P  Y  E  A  I  W  L  P  D  T  Y  V  100
                                                                                         A-loop
         ---------|---------|---------|---------|---------|---------|---------|---------|---------|---------|
 301 TACAATAGTGTGGTGATGAATCGTGAAGAGACAGAACGGTATATAAATGTGGTTATCAGCACGAACTACTGGAAAGGCGAAAGAGGAGCTGAAATAAAGT 400
 101 Y  N  S  V  V  M  N  R  E  E  T  E  R  Y  I  N  V  V  I  S  T  N  Y  W  K  G  E  R  G  A  E  I  K  F 134
                                         E-loop
         ---------|---------|---------|---------|---------|---------|---------|---------|---------|---------|
 401 TTATGTATCCAGCCTTATATCGTACCAGTTGCATGCTCGATATCAGGTTTTTCCCGTATGATCAACAAGAATGTAAGCTGATAATCTCATCATGGACCTC 500
 135  M  Y  P  A  L  Y  R  T  S  C  M  L  D  I  R  F  F  P  Y  D  Q  Q  E  C  K  L  I  I  S  S  W  T  S  167
                               Cys-loop                                                         B-loop
         ---------|---------|---------|---------|---------|---------|---------|---------|---------|---------|
 501 ATCAAAAAGCGATATCGACTATACTGCGGAGTTCCATGGTGTCAACATGGACAATTTCATTCCAAATGAAGAATGGATTGTAGTCAGCTTCAACATCAAA 600
 168  S  K  S  D  I  D  Y  T  A  E  F  H  G  V  N  M  D  N  F  I  P  N  E  E  W  I  V  V  S  F  N  I  K  200
                                       F-loop
         ---------|---------|---------|---------|---------|---------|---------|---------|---------|---------|
 601 CGGATCGAGGAGAAATTTGTCTGCTGCCCAGAACCATGGGTACTTTTGGAAGCAGTACTGGTAGTTCGACGTAAACCCCTATACTATATAGTAAATCTGG 700
 201 R  I  E  E  K  F  V  C  C  P  E  P  W  V  L  L  E  A  V  L  V  V  R  R  K  P  L  Y  Y  I  V  N  L  V 234
     C-loop                                                                                 TMD1
         ---------|---------|---------|---------|---------|---------|---------|---------|---------|---------|
 701 TCATCCCGACATCAGTCATTACAATGGTCGCCGTAACCGGTTTCTTCACCGCAGCCTCGACCAGCACTGAAAGACGTGAAAAGCTGTCCCTAGGCATCGA 800
 235  I  P  T  S  V  I  T  M  V  A  V  T  G  F  F  T  A  A  S  T  S  T  E  R  R  E  K  L  S  L  G  I  D  267
                                                                                                TMD2
         ---------|---------|---------|---------|---------|---------|---------|---------|---------|---------|
 801 CTCGTTGCTAGCCATGTCTATCCTGATGATGATGGTATCGGAGCAGATGCCAACAACCAGTGACTATGTACCTCTTTTTGGCCTATTCTATCTGACAATT 900
 268  S  L  L  A  M  S  I  L  M  M  M  V  S  E  Q  M  P  T  T  S  D  Y  V  P  L  F  G  L  F  Y  L  T  I  300
                                                                                 TMD3
         ---------|---------|---------|---------|---------|---------|---------|---------|---------|---------|
 901 ATCATTGTGATCTTCATCGGAACACTATTTACGGCAATCATACTCAATATTCACCTACAGAAGATGTACGCTCAACCAGTATCACCATTGATATCGTACT 1000
 301 I  I  V  I  F  I  G  T  L  F  T  A  I  I  L  N  I  H  L  Q  K  M  Y  A  Q  P  V  S  P  L  I  S  Y  L 334

         ---------|---------|---------|---------|---------|---------|---------|---------|---------|---------|
1001 TGTTCTTCAATAAGGTAGCCTCGTTGCTTCGATTACATCCACCGACAATGCTGTTGGAGCTCTGGGAAGAGACCGGTGTGCATTTTGGACGATCCAATCG 1100
 335  F  F  N  K  V  A  S  L  L  R  L  H  P  P  T  M  L  L  E  L  W  E  E  T  G  V  H  F  G  R  S  N  R  367

         ---------|---------|---------|---------|---------|---------|---------|---------|---------|---------|
1101 GAAGAATACACCAAAGAAAAAACCGAAGCTTCAACCTCTTAATTCTCATACCGAGCTTCTATTGAAAACGCCTCCACCGCCACTGCTGAAGACTCCTCCA 1200
 368  K  N  T  P  K  K  K  P  K  L  Q  P  L  N  S  H  T  E  L  L  L  K  T  P  P  P  P  L  L  K  T  P  P   400

         ---------|---------|---------|---------|---------|---------|---------|---------|---------|---------|
1201 CCGCCTCCATCTAGAGATTCCCCCTTCAAACAGCCGAGTATACCTGATGAAAGCACCTTGCTACGCAGTCAGACAGCTCGAAACAATTGGAAGCGTTTAG 1300
 401 P  P  P  S  R  D  S  P  F  K  Q  P  S  I  P  D  E  S  T  L  L  R  S  Q  T  A  R  N  N  W  K  R  L  A 434

         ---------|---------|---------|---------|---------|---------|---------|---------|---------|---------|
1301 CTCGGAAAGCTAGTGAACGAAGTAAAAAGGATCGCAACAATGGCAATGGTGACGTTCCACCACTTCGCCCCCTACAGCCAATTCAACCAAGTCGGCTGTC 1400
 435  R  K  A  S  E  R  S  K  K  D  R  N  N  G  N  G  D  V  P  P  L  R  P  L  Q  P  I  Q  P  S  R  L  S  467

         ---------|---------|---------|---------|---------|---------|---------|---------|---------|---------|
1401 GGTTCAGCGGGAACGAGAACAAGATGACGAGGATTCACCACCATTAATGCAATGTCTCTCGGCACTCAGCACTACTGAAAACAGTTTACTCGAAAGTAAA 1500
 468  V  Q  R  E  R  E  Q  D  D  E  D  S  P  P  L  M  Q  C  L  S  A  L  S  T  T  E  N  S  L  L  E  S  K   500

         ---------|---------|---------|---------|---------|---------|---------|---------|---------|---------|
1501 CTCAAAAGGAGGTACGCACTGGAATGGGAGTACCTGGCTACCGTCTTGGACAGACTACTGTTGATCGTCTTCTCGATAGTGGTGTTTCTCGTCACTTTCT 1600
 501 L  K  R  R  Y  A  L  E  W  E  Y  L  A  T  V  L  D  R  L  L  L  I  V  F  S  I  V  V  F  L  V  T  F  L 534
                                                       TMD4
         ---------|---------|---------|---------|---------|---------|---------|-------
1601 TGATGATCCTGATCGGCGAAGCGATGCACCTCTCATATCAAATGACATCAGATGCAGCTCATCCACTAACGCCTTAG 1677
 535  M  I  L  I  G  E  A  M  H  L  S  Y  Q  M  T  S  D  A  A  H  P  L  T  P  *   558
```
