# Supplementary figures and images for: Phylogenomics of Ligand-Gated Ion Channels Predicts Monepantel Effect
Source: PLoS Pathog. 2010 Sep 9;6(9):e1001091. doi: 10.1371/journal.ppat.1001091 (PMC2936538; doi:10.1371/journal.ppat.1001091)

Supplementary Figure 4

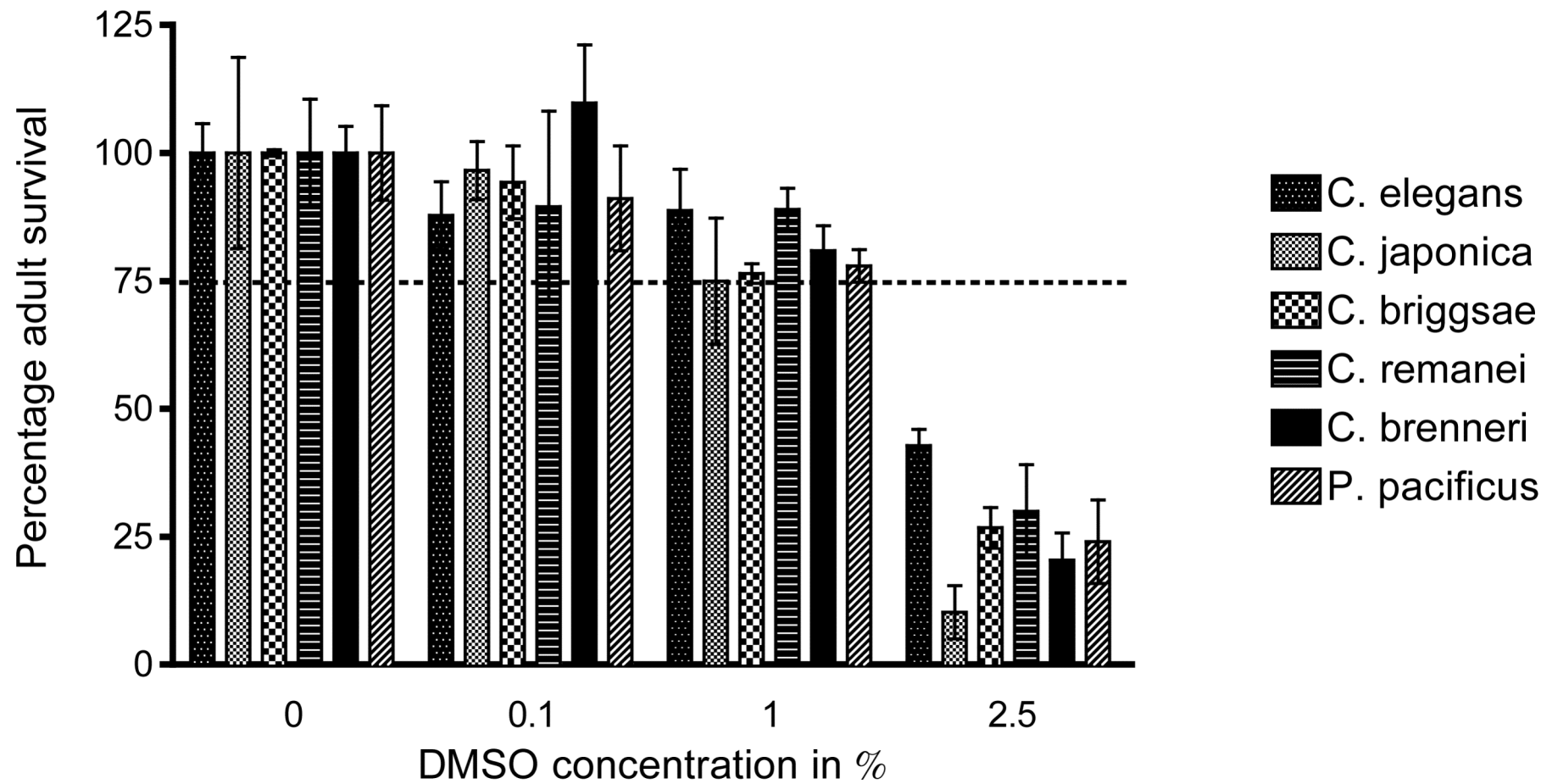

Supplement: Figure S4 — In vitro test of DMSO tolerance. Live adults on NGM wells after 3 days from egg deposition, in percent compared to control (0% DMSO) average, was plotted against DMSO concentration for Caenorhabditis elegans, C. japonica, C. briggsae, C. remanei, C. brenneri and Pristionchus pacificus. Green field background denotes presence of progeny after 6 days, indicating the ability to complete a whole life cycle. Error bars represent the SEM from three replicates. All species tolerated the typical dose of 1% DMSO. 2.5% leads to a marked development retardation. (0.29 MB PDF) [file ppat.1001091.s004.pdf]

Supplementary  
Figure 5

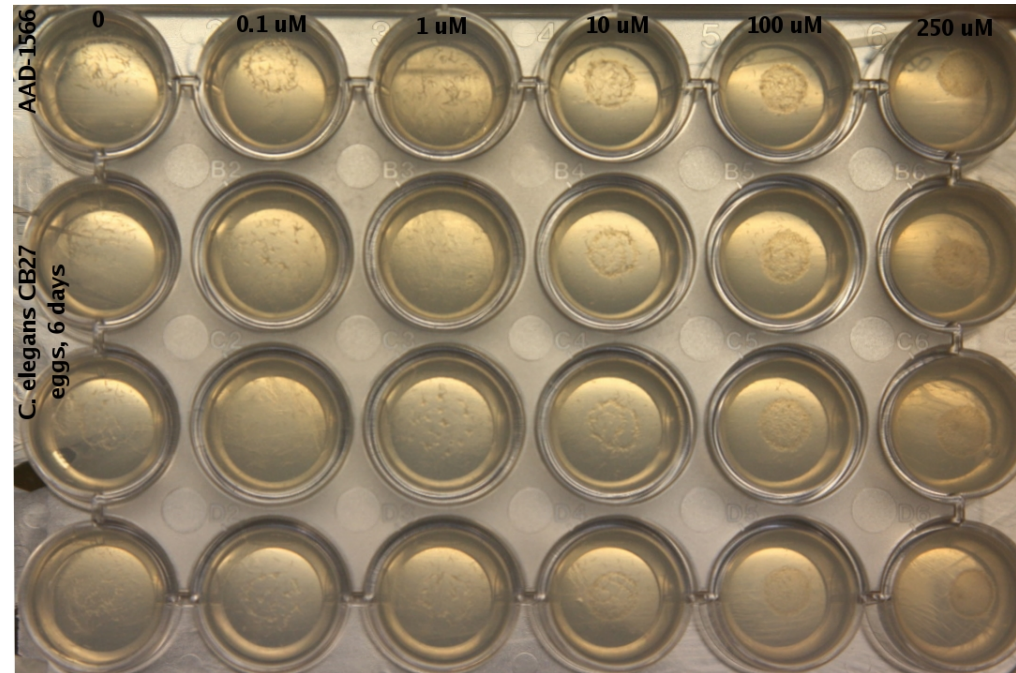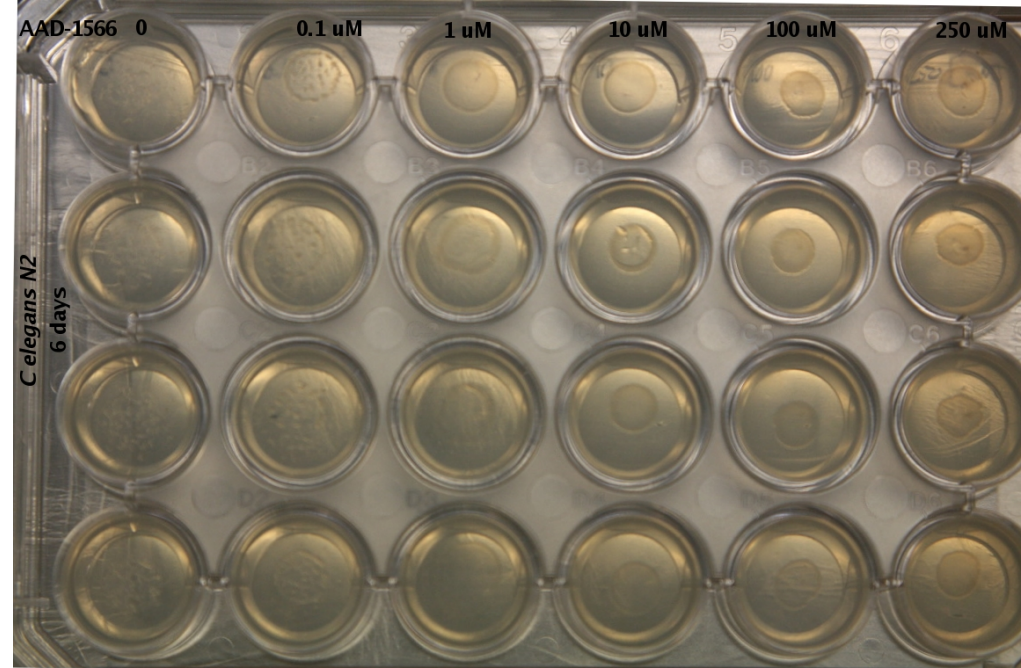

Supplement: Figure S5 — Comparison of phenotype between Cel N2 (wild type) and acr-23 (cb27) after AAD-1566 exposure. N2 worms were more sensitive, with the bacterial lawn intact down to drug doses of 1 µM, whereas feeding activity was noticeable up to 100 µM, with 1 µM being visually no more affected than the control after 6 days. (3.32 MB PDF) [file ppat.1001091.s005.pdf]

Supplementary Figure 6

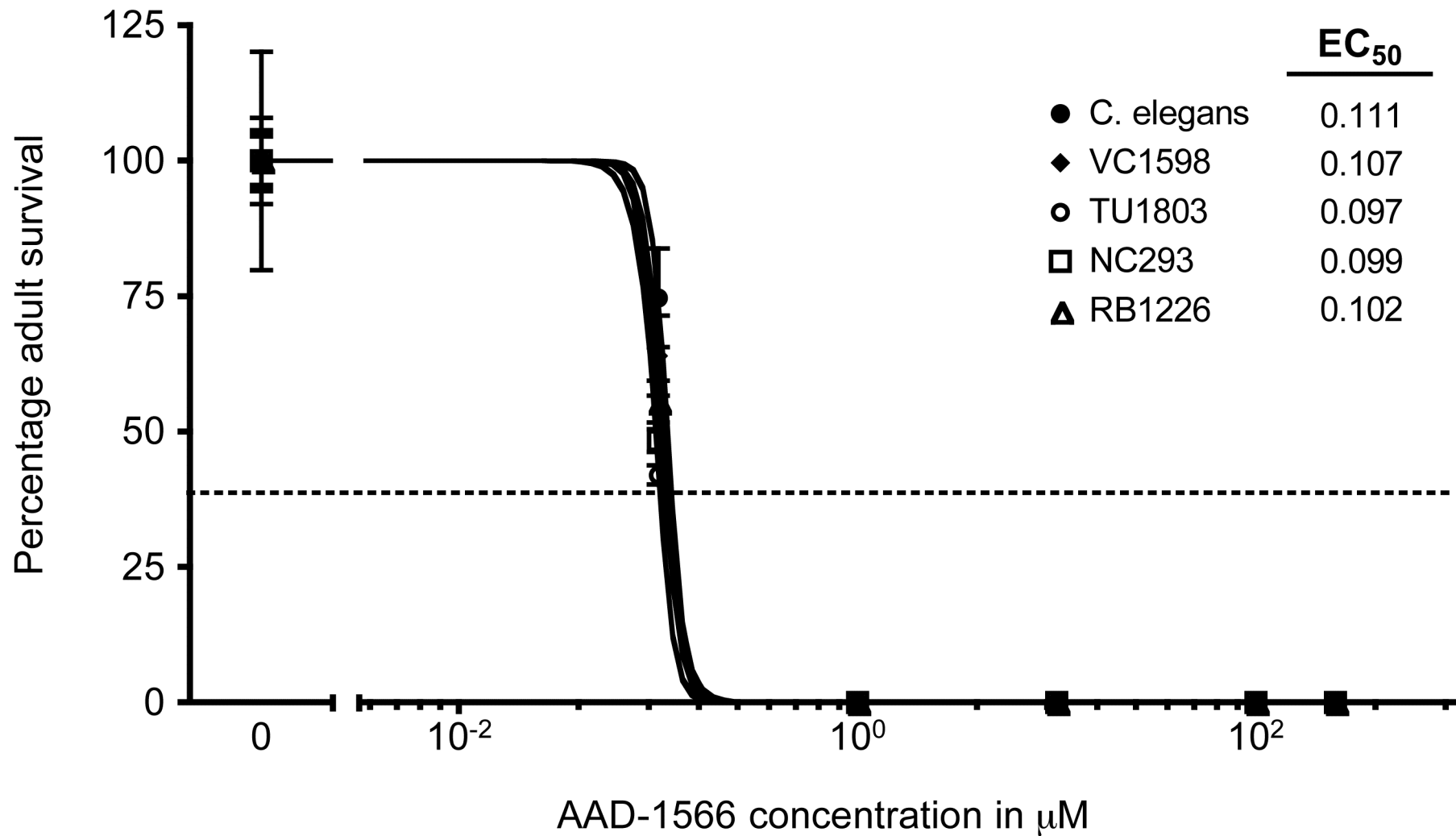

Supplement: Figure S6 — Sensitivity to AAD-1566 determined in vitro. The percentage of the average number of adult worms present after 3 days exposure relative to a control is plotted versus drug concentration for Ceanorhabditis elegans, and mutant strains VC1598, TU1803, NC293 and RB1226. Error bars represent the SEM from four replicates. Sigmoid dose-response curve fit was performed in Prism. (0.13 MB PDF) [file ppat.1001091.s006.pdf]

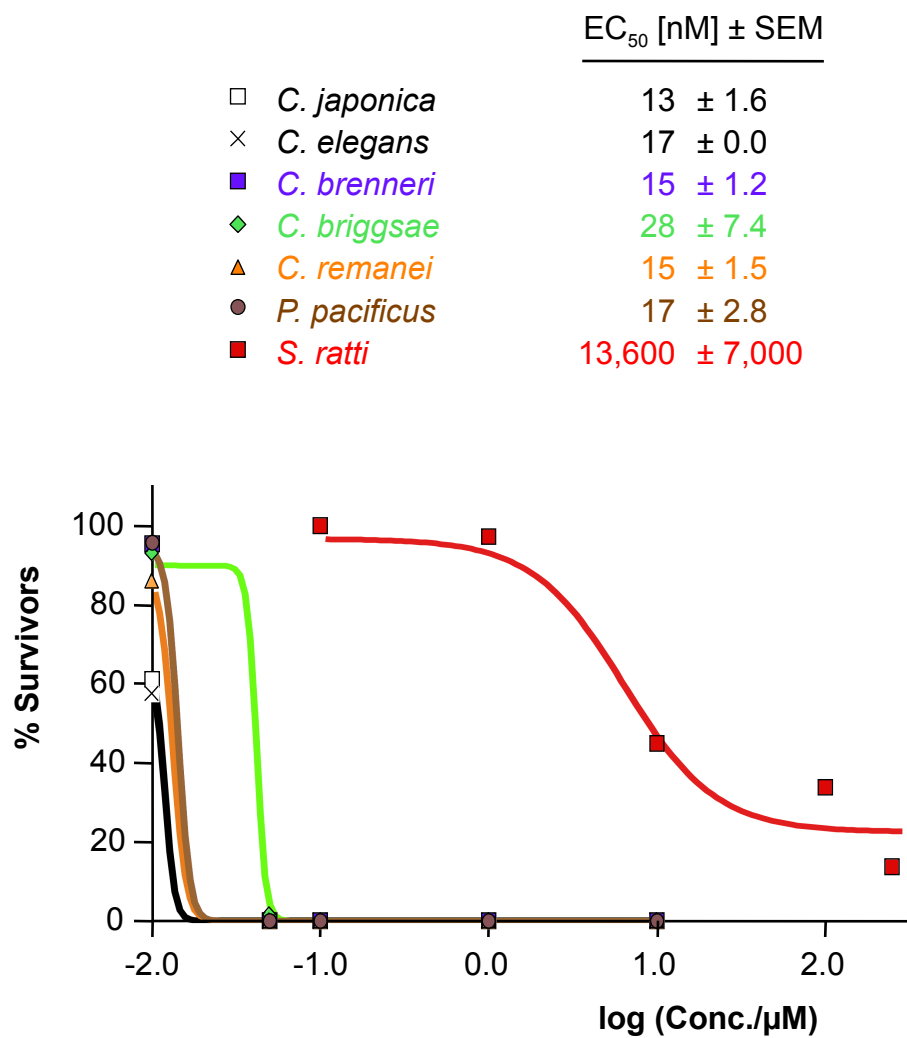

Supplement: Figure S7 — Sensitivity to ivermectin determined in vitro. The percentage of the average number of adult worms present after 3 days exposure relative to a control is plotted versus drug concentration for Caenorhabditis elegans, C. japonica, C. briggsae, C. remanei, C. brenneri and Pristionchus pacificus. Sigmoid dose-response curve fit was performed in Prism. EC50 values with standard errors, estimated from data points with four replicates, are shown inset. (0.13 MB PDF) [file ppat.1001091.s007.pdf]
