# Supplementary material for: Phylogenomics of Ligand-Gated Ion Channels Predicts Monepantel Effect
Source: PLoS Pathog. 2010 Sep 9;6(9):e1001091. doi: 10.1371/journal.ppat.1001091 (PMC2936538; doi:10.1371/journal.ppat.1001091)
Supplement: Table S1 — Primers used for PCR amplification. Primers used for PCR amplification of 18s rRNA of Caenorhabditis sp. or Pristionchus pacificus and deg-3 subfamily genes from Haemonchus contortus. (0.06 MB PDF) [file ppat.1001091.s008.pdf]

Supplementary Table 1

**Primer name**	**Sequence 5’ → 3’**
For PCR on gDNA or cDNA	
SSU18A	AAAGATTAAGCCATGCATG
SSU26R	CATTCTTGGCAAATGCTTCG
SL1	GGTTTAATTACCCAAGTTTGAG
SL2	GGTTTTAACCCAGTTACTCAAG
NheI_*acr-5*_frw2.1	GGCGGCTAGCCAATTGTCTTTACTTGGTCATGG
NotI_*acr-5*_rev2.1	GGCGGCGGCCGCTATTCGGCATCCAAGGAAGA
*Hco-acr-5*_frw10	GCAATGGTTTCCGTACGACT
*Hco-acr-5*_frw11	CAAATCCGTGGGTACTGCTC
*Hco-acr-5*_rev8	AGGAGGCATGAGCGAGATAG
*Hco-acr-5*_rev9	CTTCGACATCGACAGACGAC
NheI_*acr-17*_frw1	GGCGGCTAGCGCTATGATCAGGATGTTAGCAA
XhoI_*acr-17*_rev1	GGCGCTCGAGGGTATCAAGCGTCTGCCTTC
*Hco-acr-17*_frw1	TTGCTGTCCAAATCCATATCC
*Hco-acr-17*_frw2	CGTTACGGTGATCGGTTTCT
*Hco-acr-17*_rev4	CGATCACCGTAACGATTGTG
*Hco-acr-17*_rev5	AGTATGACGGGTTCCGTTTG
NheI_*acr-24*_frw1	GGCGGCTAGCGATGAATGAACGGGGTGAAT
XhoI_*acr-24*_rev1	GGCGCTCGAGCTAAGGCGTTAGTGGATGAGC
*Hco-acr-24*_frw1	GTTTGCTGCCCAGAACCAT
*Hco-acr-24*_frw2	CTGGTCGTTCGTCGTAAACC
*Hco-acr-24*_rev1	ACCTTTCAGTGCTGGTCGAT
*Hco-acr-24*_rev2	GGTGAAGAAGCCAGTGACG
