# Supplementary material for: Phylogenomics of Ligand-Gated Ion Channels Predicts Monepantel Effect
Source: PLoS Pathog. 2010 Sep 9;6(9):e1001091. doi: 10.1371/journal.ppat.1001091 (PMC2936538; doi:10.1371/journal.ppat.1001091)
Supplement: Table S2 — Sensitivity to DMSO determined in vitro. Number of adult worms present after 3 days exposure for C. elegans, C. japonica, C. briggsae, C. remanei, C. brenneri and P. pacificus. Green field background denotes presence of progeny after 6 days, indicating the ability to complete a whole life cycle. Yellow fields in t-test rows indicate that the hypothesis of the counts of that concentration being drawn from a normal distribution with the same average as the control (0%) could not be rejected at a 95% confidence level (two-tailed heteroscedastic t-test). (0.06 MB PDF) [file ppat.1001091.s009.pdf]

DMSO concentration in %	0	0.1	1	2.5
C. elegans	59	50	50	24
	65	65	68	29
	72	57	56	31
Average	65.3	57.3	58.0	28.0
% control	100.0	87.8	88.8	42.9
Standard deviation	6.5	7.5	9.2	3.6
t-test		0.237	0.328	0.003
C. japonica	29	30	24	0
	39	30	27	5
	20	25	15	4
Average	29.3	28.3	22.0	3.0
% control	100.0	96.6	75.0	10.2
Standard deviation	9.5	2.9	6.2	2.6
t-test		0.875	0.336	0.033
C. briggsae	71	52	65	34
	70	32	50	49
	50	41	72	48
Average	63.7	41.7	62.3	43.7
% control	100.0	65.4	97.9	68.6
Standard deviation	11.8	10.0	11.2	8.4
t-test		0.072	0.894	0.083
C. remanei	71	57	51	19
	50	36	59	29
	69	77	59	9
Average	63.3	56.7	56.3	19.0
% control	100.0	89.5	88.9	30.0
Standard deviation	11.6	20.5	4.6	10.0
t-test		0.656	0.412	0.008
C. brenneri	72	64	61	21
	78	80	51	8
	65	92	62	15
Average	71.7	78.7	58.0	14.7
% control	100.0	109.8	80.9	20.5
Standard deviation	6.5	14.0	6.1	6.5
t-test		0.494	0.057	0.000
P. pacificus	119	93	84	30
	87	74	73	35
	98	110	80	8
Average	101.3	92.3	79.0	24.3
% control	100.0	91.1	78.0	24.0
Standard deviation	16.3	18.0	5.6	14.4
t-test		0.556	0.129	0.004
