# Supplementary material for: Phylogenomics of Ligand-Gated Ion Channels Predicts Monepantel Effect
Source: PLoS Pathog. 2010 Sep 9;6(9):e1001091. doi: 10.1371/journal.ppat.1001091 (PMC2936538; doi:10.1371/journal.ppat.1001091)
Supplement: Table S3 — Sensitivity to ivermectin determined in vitro. Number of adult worms present after 3 days exposure for C. elegans, C. japonica, C. briggsae, C. remanei, C. brenneri and P. pacificus. Yellow fields in t-test rows indicate that the hypothesis of the counts of that concentration being drawn from a normal distribution with the same average as the control (0%) could not be rejected at a 95% confidence level (two-tailed heteroscedastic t-test). (0.06 MB PDF) [file ppat.1001091.s010.pdf]

Ivermectin [µM]	0	0.01	0.05	0.1	1	10
C. elegans	50	50	0	0	0	0
	50	8	0	0	0	0
	50	7	0	0	0	0
	51	50	0	0	0	0
Average	50.3	28.8	0.0	0.0	0.0	0.0
% control	100.0	57.2	0.0	0.0	0.0	0.0
Standard deviation	0.5	24.5	0.0	0.0	0.0	0.0
t-test		0.178	0.000	0.000	0.000	0.000
C. japonica	3	2	0	0	0	0
	4	4	0	0	0	0
	5	3	0	0	0	0
	6	2	0	0	0	0
Average	4.5	2.8	0.0	0.0	0.0	0.0
% control	100.0	61.1	0.0	0.0	0.0	0.0
Standard deviation	1.3	1.0	0.0	0.0	0.0	0.0
t-test		0.076	0.006	0.006	0.006	0.006
C. briggsae	50	50	1	0	0	0
	50	40	2	0	0	0
	50	50	0	0	0	0
	51	46	0	0	0	0
Average	50.3	46.5	0.8	0.0	0.0	0.0
% control	100.0	92.5	1.5	0.0	0.0	0.0
Standard deviation	0.5	4.7	1.0	0.0	0.0	0.0
t-test		0.211	0.000	0.000	0.000	0.000
C. remanei	50	50	0	0	0	0
	50	32	0	0	0	0
	50	40	0	0	0	0
	51	50	0	0	0	0
Average	50.3	43.0	0.0	0.0	0.0	0.0
% control	100.0	85.6	0.0	0.0	0.0	0.0
Standard deviation	0.5	8.7	0.0	0.0	0.0	0.0
t-test		0.195	0.000	0.000	0.000	0.000
C. brenneri	50	45	0	0	0	0
	50	50	0	0	0	0
	50	50	0	0	0	0
	51	46	0	0	0	0
Average	50.3	47.8	0.0	0.0	0.0	0.0
% control	100.0	95.0	0.0	0.0	0.0	0.0
Standard deviation	0.5	2.6	0.0	0.0	0.0	0.0
t-test		0.152	0.000	0.000	0.000	0.000
P. pacificus	33	27	0	0	0	0
	22	20	0	0	0	0
	30	33	0	0	0	0
	36	36	0	0	0	0
Average	30.3	29.0	0.0	0.0	0.0	0.0
% control	100.0	95.9	0.0	0.0	0.0	0.0
Standard deviation	6.0	7.1	0.0	0.0	0.0	0.0
t-test		0.797	0.002	0.002	0.002	0.002
