# Supplementary material for: Phylogenomics of Ligand-Gated Ion Channels Predicts Monepantel Effect
Source: PLoS Pathog. 2010 Sep 9;6(9):e1001091. doi: 10.1371/journal.ppat.1001091 (PMC2936538; doi:10.1371/journal.ppat.1001091)
Supplement: Table S4 — Sensitivity to AAD-1566 determined in vitro. Number of adult worms present after 3 days exposure for C. elegans, C. japonica, C. briggsae, C. remanei, C. brenneri and P. pacificus. Green field background denotes presence of progeny after 6 days, indicating the ability to complete a whole life cycle. Yellow fields in t-test rows indicate that the hypothesis of the counts of that concentration being drawn from a normal distribution with the same average as the control (0%) could not be rejected at a 95% confidence level (two-tailed heteroscedastic t-test). (0.06 MB PDF) [file ppat.1001091.s011.pdf]

AAD-1566 [µM]	0	0.1	1	10	100	250
C. elegans	43	40	0	0	0	0
	53	44	0	0	0	0
	48	52	0	0	0	0
	52	39	0	0	0	0
Average	49.0	43.8	0.0	0.0	0.0	0.0
% control	100.0	89.3	0.0	0.0	0.0	0.0
Standard deviation	4.5	5.9	0.0	0.0	0.0	0.0
t-test		0.212	0.000	0.000	0.000	0.000
C. japonica	61	2	0	0	0	0
	54	3	1	0	0	0
	42	2	2	0	0	0
	50	0	0	0	0	0
Average	51.8	1.8	0.8	0.0	0.0	0.0
% control	100.0	3.4	1.4	0.0	0.0	0.0
Standard deviation	7.9	1.3	1.0	0.0	0.0	0.0
t-test		0.001	0.001	0.001	0.001	0.001
C. briggsae	47	49	9	0	0	0
	59	52	20	0	0	0
	53	39	24	0	0	0
	47	34	7	0	0	0
Average	51.5	43.5	15.0	0.0	0.0	0.0
% control	100.0	84.5	29.1	0.0	0.0	0.0
Standard deviation	5.7	8.4	8.3	0.0	0.0	0.0
t-test		0.174	0.001	0.000	0.000	0.000
C. remanei	34	29	26	0	0	0
	30	26	20	1	0	0
	39	18	16	2	0	0
	33	23	11	2	0	0
Average	34.0	24.0	18.3	1.3	0.0	0.0
% control	100.0	70.6	53.7	3.7	0.0	0.0
Standard deviation	3.7	4.7	6.3	1.0	0.0	0.0
t-test		0.017	0.008	0.000	0.000	0.000
C. brenneri	70	53	16	8	2	0
	62	57	24	12	9	1
	70	31	28	16	6	0
	61	46	16	8	3	0
Average	65.8	46.8	21.0	11.0	5.0	0.3
% control	100.0	71.1	31.9	16.7	7.6	0.4
Standard deviation	4.9	11.4	6.0	3.8	3.2	0.5
t-test		0.037	0.000	0.000	0.000	0.000
P. pacificus	50	66	26	20	23	0
	62	49	49	36	33	1
	50	47	38	37	12	2
	44	63	36	15	9	1
Average	51.5	56.3	37.3	27.0	19.3	1.0
% control	100.0	109.2	72.3	52.4	37.4	1.9
Standard deviation	7.5	9.6	9.4	11.2	11.0	0.8
t-test		0.469	0.058	0.014	0.004	0.001
CB27	126	114	171	127	82	40
	154	112	142	93	69	56
	176	170	108	93	58	50
	179	133	158	102	77	20
Average	158.8	132.3	144.8	103.8	71.5	41.5
% control	100.0	256.8	281.1	201.5	138.8	80.6
Standard deviation	24.5	26.9	27.2	16.1	10.5	15.8
t-test		0.196	0.474	0.012	0.003	0.000
