# Supplementary material for: Phylogenomics of Ligand-Gated Ion Channels Predicts Monepantel Effect
Source: PLoS Pathog. 2010 Sep 9;6(9):e1001091. doi: 10.1371/journal.ppat.1001091 (PMC2936538; doi:10.1371/journal.ppat.1001091)
Supplement: Table S5 — Sensitivity to AAD-1566 determined in vitro for mutant C. elegans isolates VC1598: Cel-acr-20(ok1849)/mT1 II; +/mT1[dpy-10(e128)] III, NC293: Cel-acr-5(ok180) III, TU1803: Cel-deg-3(u662) Cel-des-2(u695) V and RB1226: Cel-acr-18(ok1285) V. Green field background denotes presence of progeny after 6 days, indicating the ability to complete a whole life cycle. Yellow fields in t-test rows indicate that the hypothesis of the counts of that concentration being drawn from a normal distribution with the same average as the control (0%) could not be rejected at a 95% confidence level (two-tailed heteroscedastic t-test). (0.05 MB PDF) [file ppat.1001091.s012.pdf]

AAD-1566 [µM]	0	0.1	1	10	100	250
C. elegans	129	55	0	0	0	0
	88	81	0	0	0	0
	107	98	0	0	0	0
	119	97	0	0	0	0
Average	110.8	82.8	0.0	0.0	0.0	0.0
% control	100.0	74.7	0.0	0.0	0.0	0.0
Standard deviation	17.6	20.1	0.0	0.0	0.0	0.0
t-test		0.082	0.001	0.001	0.001	0.001
**VC1598**	32	19	0	0	0	0
	27	12	0	0	0	0
	12	12	0	0	0	0
	18	14	0	0	0	0
Average	22.3	14.3	0.0	0.0	0.0	0.0
% control	100.0	64.0	0.0	0.0	0.0	0.0
Standard deviation	9.0	3.3	0.0	0.0	0.0	0.0
t-test		0.173	0.016	0.016	0.016	0.016
**TU1803**	113	38	0	0	0	0
	103	42	0	0	0	0
	102	46	0	0	0	0
	89	45	0	0	0	0
Average	101.8	42.8	0.0	0.0	0.0	0.0
% control	100.0	42.0	0.0	0.0	0.0	0.0
Standard deviation	9.8	3.6	0.0	0.0	0.0	0.0
t-test		0.000	0.000	0.000	0.000	0.000
**NC293**	58	26	0	0	0	0
	56	32	0	0	0	0
	68	31	0	0	0	0
	64	30	0	0	0	0
Average	61.5	29.8	0.0	0.0	0.0	0.0
% control	100.0	48.4	0.0	0.0	0.0	0.0
Standard deviation	5.5	2.6	0.0	0.0	0.0	0.0
t-test		0.000	0.000	0.000	0.000	0.000
**RB1226**	57	34	0	0	0	0
	70	42	0	0	0	0
	75	32	0	0	0	0
	68	42	0	0	0	0
Average	67.5	37.5	0.0	0.0	0.0	0.0
% control	100.0	55.6	0.0	0.0	0.0	0.0
Standard deviation	7.6	5.3	0.0	0.0	0.0	0.0
t-test		0.001	0.000	0.000	0.000	0.000
